# Pyruvate kinase M2 activation maintains mitochondrial metabolism by regulating the interaction between HIF-1α and PGC-1α in diabetic kidney disease

**DOI:** 10.1186/s10020-025-01320-4

**Published:** 2025-07-25

**Authors:** Jimin Park, Young Su Joo, Bo Young Nam, Gyuri Kim, Jung Tak Park, Tae-Hyun Yoo, Shin-Wook Kang, Seung Hyeok Han

**Affiliations:** 1 Department of Internal Medicine, College of Medicine, Institute of Kidney Disease Research, Yonsei University, 50-1 Yonsei-ro, Seodaemun-gu, Seoul, 03722 Republic of Korea; 2 Severance Biomedical Science Institute, College of Medicine, Yonsei University, Seoul, Korea; 3 Department of Biochemistry and Molecular Biology, Indiana University School of Medicine, Indianapolis, IN USA; 4 Division of Nephrology, Department of Internal Medicine, Yongin Severance Hospital, Yonsei University College of Medicine, Yongin, Korea

**Keywords:** Pyruvate kinase M2, Mitochondrial metabolism, Diabetic kidney disease

## Abstract

**Background:**

Pyruvate kinase isoform M2 (PKM2) activation has been suggested as a potential protective mechanism against kidney injury by improving mitochondrial dysfunction and anaerobic glycolysis. However, the underlying molecular mechanisms are unclear. Herein, we have demonstrated that PKM2 activation alleviates HIF-1α-mediated suppression of PGC-1α in diabetic kidney disease (DKD) models.

**Methods:**

In animal DKD study, *db*/*db* mice were intraperitoneally injected with TEPP-46, a PKM2 activator. In vitro, primary cultured renal tubular epithelial cells (RTECs) from C57BL/6 mice were exposed to high glucose (HG) conditions with and without TEPP-46. The interaction between HIF-1α and PGC-1α was investigated using HIF-1α overexpression and suppression.

**Results:**

Our findings in *db/db* mice kidneys unveiled a reduced PKM2 activation, aberrant glycolysis, impaired fatty acid oxidation, and decreased mitochondrial mass, integrity, and function under diabetic conditions. These changes were accompanied by increased HIF-1α and decreased PGC-1α levels. Furthermore, diabetic kidney exhibited increased fibrosis and apoptosis markers. Notably, direct PKM2 activation by TEPP-46 treatment counteracted the perturbed energy metabolism, restored mitochondrial function, and reduced cell death. Similar effects were also observed in HG-treated RTECs upon TEPP-46 intervention. Mechanistically, our chromatin immunoprecipitation assay revealed that HIF-1α directly bound to the regulatory region of the *Ppargc1a* promoter, and this interaction was inversely dependent on PKM2 activation. Moreover, *Hif1ɑ* overexpression suppressed *Ppargc1a* and triggered aberrant energy metabolism, mitochondrial dysfunction, and apoptosis. These changes were reversed by HIF-1α suppression.

**Conclusion:**

Our study highlights the role of PKM2 activation in restoring impaired mitochondrial metabolism and function by modulating HIF-1α and PGC-1α interactions in DKD.

**Supplementary Information:**

The online version contains supplementary material available at 10.1186/s10020-025-01320-4.

## Introduction

Diabetic kidney disease (DKD) is the leading cause of kidney failure with replacement therapy (KFRT) (Bowe et al. 2018; Jin et al. 2015). Approximately 40% of patients with type 2 diabetes develop DKD, and 10% of these patients eventually undergo KFRT (American Diabetes Association 2010; Retnakaran et al. 2006). Hence, it is important to find effective therapeutic targets to prevent DKD. In diabetes, metabolic dysregulation caused by hyperglycemia induces alterations in the kidney, including glomerular hypertrophy, mesangial expansion, basement membrane thickening, and tubulointerstitial inflammation and fibrosis (Thomas et al. 2015). While glomerular changes in structure and function are integral aspects of DKD, diabetes-induced tubular injury has recently been recognized as an important contributor to DKD. Renal tubular epithelial cells (RTECs) are highly metabolically active and energy-demanding cells with abundant mitochondria (O’Connor 2006; Weidemann et al. 1969). Many studies have demonstrated that mitochondrial dysfunction contributes to DKD, which is characterized by reduced peroxisome proliferator-activated receptor-γ coactivator-1α (PGC-1α) activity, a key regulator of mitochondrial biogenesis, altered mitochondrial dynamics, and suppressed TCA cycle and fatty acid oxidation (FAO) (Forbes et al. 2018; Han et al. 2017; Sharma et al. 2013). RTECs favor gluconeogenesis with negligible glycolysis under physiological conditions (Guder et al. 1992; Uchida and Endou 1988). However, in poorly oxygenated environments, the metabolic flux of glucose can shift from oxidative phosphorylation in the mitochondria to lactate fermentation by lactate dehydrogenase, resulting in inefficient ATP production (Kierans et al. 2021; Warburg 1956). This aberrant glycolytic flux, called the “Warburg effect,” was originally reported in cancer metabolism but can be also observed in DKD (Sas et al. 2016).

Pyruvate kinase isoform M2 (PKM2) is a rate-limiting enzyme that catalyzes the last step of glycolysis in which phosphoenolpyruvate is converted to pyruvate, the end-product of glycolysis. Pyruvate is then converted to acetyl coenzyme A (acetyl-CoA) by pyruvate dehydrogenase, which enters the TCA cycle. The conformational changes of PKM2 act as a “nutritional sensor and growth signaling switch,” which allows cells to adapt to a fluctuating nutritional microenvironment (Israelsen et al. 2015; Mazurek 2011; Morgan et al. 2013). Interestingly, a previous study demonstrated a role of PKM2 in podocyte injury under diabetic conditions (Qi et al. 2017). This study found that hyperglycemia led to a reduction in PKM2 tetramer formation. Additionally, a podocyte-specific knockout of *Pkm2* in diabetic mice resulted in increased albuminuria, exacerbated glomerular pathology, and aggravated mitochondrial dysfunction. Conversely, pharmacologically induced stabilization of the PKM2 tetramer reduced toxic metabolites and improved mitochondrial dysfunction by restoring glycolytic flux and *Ppargc1a* mRNA levels. However, the study did not clearly elucidate the precise mechanism responsible for the increased PGC-1α levels caused by PKM2 activation.

Given that PKM2 and PGC-1α play a major role in mitochondrial energy and metabolism, exploring the intricate link between the glycolytic sensor and mitochondrial regulator holds great promise. Thus, we undertook this study to unravel the mechanisms underlying how PKM2 activation regulates PGC-1α in high glucose (HG)-stimulated RTECs and mice with type 2 diabetes.

## Research design and methods

Additional details for all methods are provided in the Electronic Supplementary Material and Table S1.

### Animal study and treatment

For the animal study of type 2 diabetes, we purchased B6.Cg-M+/+Lepr^db^/Lepr^db^ (*db/db*) male mice and their heterozygous littermates B6.Cg-M+/-Lepr^db^/+ (*db/m*) as non-diabetic controls from Jackson Laboratories (Bar Harbor, ME, USA). Animals were randomized to treatment groups to minimize bias. Ten male mice each in the diabetes and control groups were intraperitoneally injected with TEPP-46 (10 mg/kg) or dimethyl sulfoxide, respectively, at 6 weeks of age for 6 weeks. After 6 weeks, the kidneys were extracted under anesthesia induced with Zoletil (10 mg/kg) (Virbac, Carros, France), after which the animals were sacrificed. Kidney samples were immediately frozen in liquid nitrogen and stored at −70 °C until use. The blood samples were collected via the left ventricle using a 23-gauge needle (Parasuraman et al. 2010).

### Primary culture of RTECs and transfection

Mouse RTECs were isolated from C57BL/6 male mice. At a confluency of approximately 70%, the RTECs were FBS-restricted for 24 h, and the medium was replaced with 1% FBS DMEM medium for the control group and with D-glucose (40 mM) to induce an HG environment. RTECs were harvested 48 h after medium changes. We used TEPP-46, a small-molecule PKM2 activator (Selleck Chemicals, Houston, TX, USA), to induce PKM2 activation (Anastasiou et al. 2012). Based on a previous study, we treated cells with 10 µM TEPP-46 in all experiments (Qi et al. 2017). In *Hif1a* and *Pkm2* suppression experiments, RTECs were transfected with a mouse *Hif1a* small interfering RNA plasmid (siRNA), *Pkm2* siRNA (GE Dharmacon, Buckinghamshire, UK), or both using Lipofectamine RNAiMAX reagents (Invitrogen, Carlsbad, CA, USA), and media were changed to serum-free media after 6 h of transfection. For *Hif1a* overexpression, cells were transfected with mouse *Hif1a* plasmid (1 µg) or vector plasmid (Addgene, Cambridge, MA, USA) using Lipofectamine 3000 and Plus reagents (Invitrogen, Carlsbad, CA, USA). The cells were then incubated for an additional 48 h and analyzed.

### Cross-linking for determination of PKM2 isomers

To assess the status of PKM2 subunit association, we conducted a disuccinimidyl suberate (DSS)-mediated cross-linking study. RTECs were lysed in phosphate-buffered saline (PBS) with 0.5% Triton X-100 (Sigma-Aldrich, St. Louis, MO, USA) and scraped from culture plates. Cells from kidney tissue samples were lysed in PBS with 0.5% Triton X-100 and a protease inhibitor (Thermo Fisher Scientific, Waltham, MA, USA) for 30 min at 4℃. Fresh DSS (2 mM) (Thermo) was then added to the samples and incubated for 30 min at 37℃. A quenching solution with 1 M Tris was added to the samples and incubated for 15 min at room temperature. The cross-linked samples were added to 4X NuPAGE LDS sample buffer (Thermo) and boiled for 5 min at 100℃. The samples were then separated by 4–12% Bis-Tris gradient gel (Sigma-Aldrich) and transferred to polyvinylidene difluoride membranes. The membranes were incubated with 0.4% paraformaldehyde in PBS at room temperature for 30 min. PKM2 antibody (Cell Signaling Technology, Danvers, MA, USA) was then added for detection of PKM2 tetramers, dimers, and monomers.

### Chromatin immunoprecipitation assay

A chromatin immunoprecipitation (ChIP) assay was conducted as previously described (Sun et al. 2010). Briefly, 2 × 10^7^ primary culture cells were cross-linked, washed, and sonicated. The resulting lysates were subjected to immunoprecipitation with antibodies against mouse HIF-1α (Abcam, Cambridge, MA, USA) or control IgG (Santa Cruz Biotechnology, Santa Cruz, CA, USA). Protein A agarose/salmon sperm DNA (Millipore, Temecula, CA, USA) was used to capture the immunoprecipitants. Bound proteins were eluted after washing, and ChIP-enriched DNA was isolated by phenol: chloroform extraction. The eluted ChIP DNA and input control samples were analyzed by qPCR using the following primer pair within the *Ppargc1a* enhancer promoter: the primer sequences were sense 5’-CACGTGTGATGTAGCTGGTGCAG-3’ and anti-sense 5’- CTTGCATTCCATTCCTTACACGT-3’. Data were normalized to the input samples as described elsewhere (Sun et al. 2010).

## Results

### PKM2 activation by TEPP-46 attenuates altered energy metabolism

After the 6-week experimental period, the body and kidney weights increased in diabetic mice compared with control mice (Table S2). Significant increases in serum glucose, blood urea nitrogen (BUN), and creatinine levels were observed in diabetic mice compared with control mice. TEPP-46 treatment did not influence the body weight, kidney weight, or serum glucose level at sacrifice. There is no significant difference in glucose levels between the control group and the TEPP-46 treatment group during the experiment period (Fig S1). Compared to control diabetic mice, TEPP-46-treated mice exhibited significantly lower BUN and creatinine levels. The 24-hour urine albumin excretion at sacrifice was significantly higher in diabetic mice than in control mice, and TEPP-46 treatment reduced albuminuria.

**Fig. 1:**
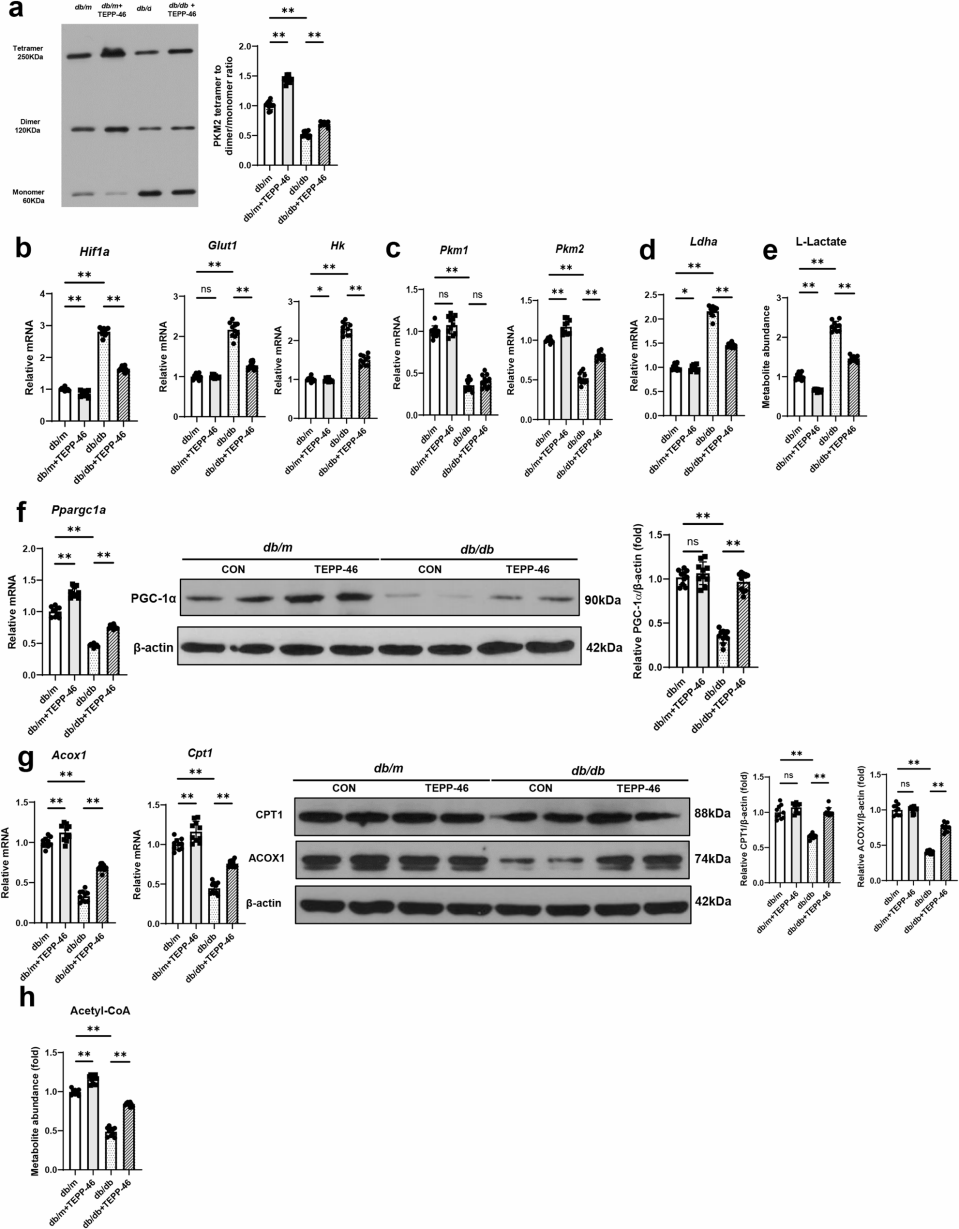
PKM2 activation by TEPP-46 treatment restores altered glycolysis, PGC-1α expression, and fatty acid oxidation in *db/db* mice. Both *db/db* and *db/m* mice were treated with TEPP-46 (10 mg/kg) or dimethyl sulfoxide (20 µL) via intraperitoneal injection for 6 weeks. **a** The PKM2 tetramer to dimer/monomer ratios were lower in *db/db* mice in a disuccinimidyl suberate-mediated cross-linking study. **b**-**d** The mRNA expression levels of genes of the glycolysis pathway were altered, and **e** the concentrations of L-lactate were increased in *db/db* mice. **f** The mRNA expression levels of *Ppargc1a* and the corresponding protein levels of PGC-1α were reduced in *db/db* mice, which were restored upon TEPP-46 treatment. **g** The mRNA and protein expression levels of CPT1 and ACOX1, markers for fatty acid oxidation, were reduced in *db/db* mice and were increased upon TEPP-46 treatment. **h** The acetyl-CoA levels measured using a colorimetric assay were decreased in *db/db* mice, and the decreased levels were restored by TEPP-46 treatment. For all groups, data are presented as mean ± SD (*n* = 10 per group). ns, *P* ≥ 0.05; *, *P* < 0.05; **, *P* < 0.01

Because PKM2 tetramers exhibit more potent pyruvate kinase enzyme activity than PKM2 dimers and monomers, the PKM2 activation state can be determined by examining the shift in PKM2 tetramer and dimer/monomer distribution. Therefore, we assessed the changes in expression levels of PKM2 subunits via a DSS cross-linking study. The ratios of the PKM2 tetramers to the PKM2 dimers and monomers were lower in *db/db* mice than in *db/m* mice. The decreased ratios were significantly increased by TEPP-46 treatment (Fig. 1a).

Since PKM2 acts as a glycolytic sensor, we examined the effects of TEPP-46 treatment on the glycolytic pathway in *db/db* mice. The mRNA levels of genes involved in the glycolytic pathway, such as *Glut1*, *Hk*, *Ldha*, and *Hif1a*, were higher in diabetic mice than in control mice, whereas *Pkm2* expression levels decreased, suggesting diversion of glycolytic flux into lactate production (Fig. 1b and d). This finding was confirmed by a significant accumulation of L-lactate in *db/db* mice than in control mice (Fig. 1e). These alterations were reversed by TEPP-46 treatment, suggesting improvements in the aberrant glycolytic flux. However, TEPP-46 did not affect the decreased transcript level of *Pkm1* in diabetic mice (Fig. 1c). PGC-1α regulates mitochondrial biogenesis and FAO metabolism. Therefore, we examined PGC-1α expression levels in diabetic mice with and without a PKM2 activator. The mRNA and protein levels of PGC-1α were significantly lower in *db/db* mice (Fig. 1f). TEPP-46 treatment increased the reduced expression levels of PGC-1α in the diabetic kidney. Additionally, the FAO pathway was also suppressed, evidenced by decreased transcript and protein levels of CPT1 and ACOX1 (Fig. 1g). These alterations were significantly restored by PKM2 activation. Next, we examined acetyl-CoA levels using a colorimetric assay. Acetyl-CoA is generated by both glycolysis and FAO, after which it enters the TCA cycle. As these two pathways were dysregulated in *db/db* mice, the acetyl-CoA concentration significantly decreased. The reduced acetyl-CoA concentration was restored by TEPP-46 treatment (Fig. 1h).

An in vitro study recapitulated the findings of the animal study (Fig. S2). HG-stimulated RTECs displayed dysregulated energy metabolism, which was reversed by TEPP-46 treatment. PKM2 and PGC-1α downregulation were not induced by 24 mM mannitol treatment, suggesting that PKM2 activity is influenced solely by glucose levels and not by osmotic pressure (Fig S3). To better reflect the glucose concentrations observed in diabetic animal models, we repeated the key experiments using 30 mM glucose, which corresponds to the upper pathological range of blood glucose levels typically observed in diabetic mice (approximately 500–540 mg/dL) (Fig. S4). The results closely mirrored those obtained under 40 mM glucose conditions, thereby reinforcing the pathophysiological relevance of our in vitro findings in the context of animal study.

Collectively, these findings suggest that diabetes can cause aberrant glycolytic flux, decrease PGC-1α expression, and suppress the FAO pathway. PKM2 activation by TEPP-46 can significantly improve the altered metabolism.

### HIF-1α directly regulates PGC-1α in response to PKM2

Previous studies demonstrated that PKM2 was the key determinant for stabilizing HIF-1α and regulating *Hif1a*-dependent genes (Luo et al. 2011; Palsson-McDermott et al. 2015), Additionally, there is evidence that PKM2 activation by TEPP-46 inhibited lipopolysaccharide (LPS)-induced HIF-1α and the expression of other *Hif1a*-dependent genes (Palsson-McDermott et al. 2015). Similarly, we demonstrated that HIF-1α levels increased in diabetic conditions, and this increase was reversed by PKM2 activation with TEPP-46. Interestingly, in clear renal cell carcinoma, PGC-1α is suppressed by an HIF-1α/Dec1-dependent mechanism (LaGory et al. 2015). Therefore, we hypothesized that HIF-1α may directly regulate PGC-1α in response to PKM2 activity in RTECs.

The regulation of PKM2 on PGC-1α via HIF-1α was investigated by analyzing changes in the expression levels of HIF-1α and PGC-1α in response to the silencing of *Hif1a*,* Pkm2*, or both *Hif1a* and *Pkm2* under HG conditions (Fig. S5). Silencing *Pkm2* significantly increased the mRNA expression of *Hif1a*, with these changes being more pronounced in HG-treated RTECs. The mRNA expression level of *Hif1a* in RTECs with double silencing of *Pkm2* and *Hif1a* was similar to those with *Hif1a* silencing alone (Fig. S5a). Inverse changes in both the mRNA expression and protein levels of PGC-1α were observed in response to the single or double silencing of *Pkm2* or *Hif1a* (Fig. S5b). The transcript levels of *Cpt1* and *Acox1* showed similar relationships with PGC-1α activity in response to the suppression of *Pkm2*,* Hif1a*, or both (Fig. S5c). These data suggest that HIF-1α is a downstream mediator of PKM2, playing a key role in the regulatory effects of PKM2 on PGC-1α.

**Fig. 2:**
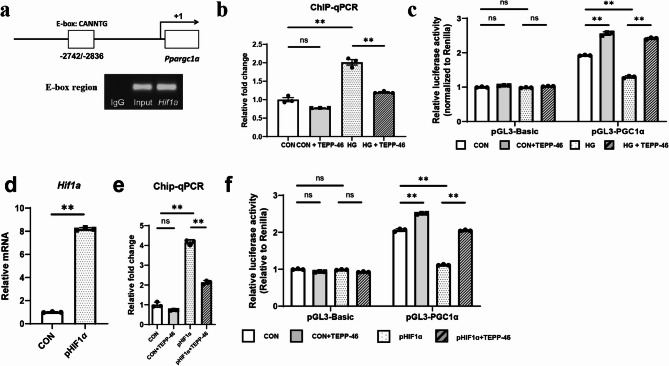
*Hif1a* directly inactivates *Ppargc1a* in renal tubular epithelial cells (RTECs). **a-c **Primary RTECs were stimulated with 40 mM glucose (HG) and 10 µM TEPP-46 for 48 h. **a** chromatin immunoprecipitation (ChIP) assay using a primer spanning the putative hypoxia-responsive element region (HRE) within the E-box flanking region of the *Ppargc1a* promoter. **b** In the ChIP assay, HG-treated RTECs with HG showed an increased enrichment of the HRE binding region in *Ppargc1a*. **c** Luciferase reporter activity assay using RTECs from primary culture after transfection of the pGL3-PGC-1α plasmid. *Ppargc1a* promoter-driven luciferase reporter activity was significantly lower in HG-treated RTECs. These alterations were reversed by TEPP-46 treatment. **d-f** Primary RTECs were transfected with a *Hif1a* plasmid (pHIF1α) or vector plasmid and 10 µM TEPP-46 for 48 h. **d** The mRNA expression levels of *Hif1a* were elevated in pHIF1α-transfected RTECs. **e** In the ChIP assay, transfection of RTECs with pHIF1α increased the enrichment of the HRE binding region in the *Ppargc1a* promoter compared with controls. **f** Luciferase reporter activity assay using RTECs from primary culture after co-transfection of pGL3-PGC-1α and pHIF1α plasmids. *Ppargc1a* promoter-driven luciferase reporter activity was significantly lower in *Hif1a*-transfected RTECs. These alterations were reversed by TEPP-46 treatment. For all groups, data are presented as mean ± SD (*n* = 3 per group). ns, *P* ≥ 0.05; *, *P* < 0.05; **, *P* < 0.01; CON, control

To examine the interaction between HIF-1α and PGC-1α, we performed a ChIP assay using primers spanning the putative hypoxia-responsive element (HRE) region within the E-box flanking region of the *Ppargc1a* mouse gene (Fig. 2a). The enrichment of the HRE binding region in the *Ppargc1a* promoter significantly increased in HG-treated RTECs, whereas it decreased by TEPP-46 co-treatment (Fig. 2b). To substantiate this finding, luciferase reporter assay was also performed. *Ppargc1a* promoter-driven luciferase reporter activity was significantly reduced in HG-treated RTECs, while TEPP-46 co-treatment significantly reversed this activity (Fig. 2c). To further clarify the mechanistic link between HIF-1α and PGC-1α, we directly overexpressed *Hif1a* using the mouse *Hif1a* plasmid (pHIFα) with or without TEPP-46. The plasmid transfection robustly induced *Hif1a* gene expression in RTECs (Fig. 2d). The ChIP assay showed that *Hif1a*-overexpressing RTECs increased the enrichment of the HRE binding region in the *Ppargc1a* promoter, and *Ppargc1a*-luciferase reporter activity was reduced by *Hif1a* overexpression (Fig. 2e and f). This suppressed PGC-1α activity was alleviated by PKM2 activation with TEPP-46 treatment. To determine whether PKM2 activation regulates *Ppargc1a* expression independently of HIF-1α, we performed siRNA-mediated knockdown of *Hif1a* in HG–stimulated RTECs, with or without TEPP-46 treatment (Fig. S6). Silencing *Hif1a* led to a partial restoration of *Ppargc1a* expression. Notably, TEPP-46 further enhanced *Ppargc1a* levels even in the absence of HIF-1α, suggesting that PKM2 activation may also regulate mitochondrial metabolism through HIF-1α–independent mechanisms.

Collectively, these data suggest that *Hif1a* directly binds to an HRE within the *Ppargc1a* promoter region and inhibits its transcription, and HIF-1α acts on PGC-1α in a PKM2-dependent manner. However, this regulation is not solely mediated by HIF-1α, indicating the involvement of additional PKM2-dependent, HIF-1α–independent pathways.

### PKM2 activation attenuates altered mitochondrial biogenesis, dynamics, morphology, and function

**Fig. 3:**
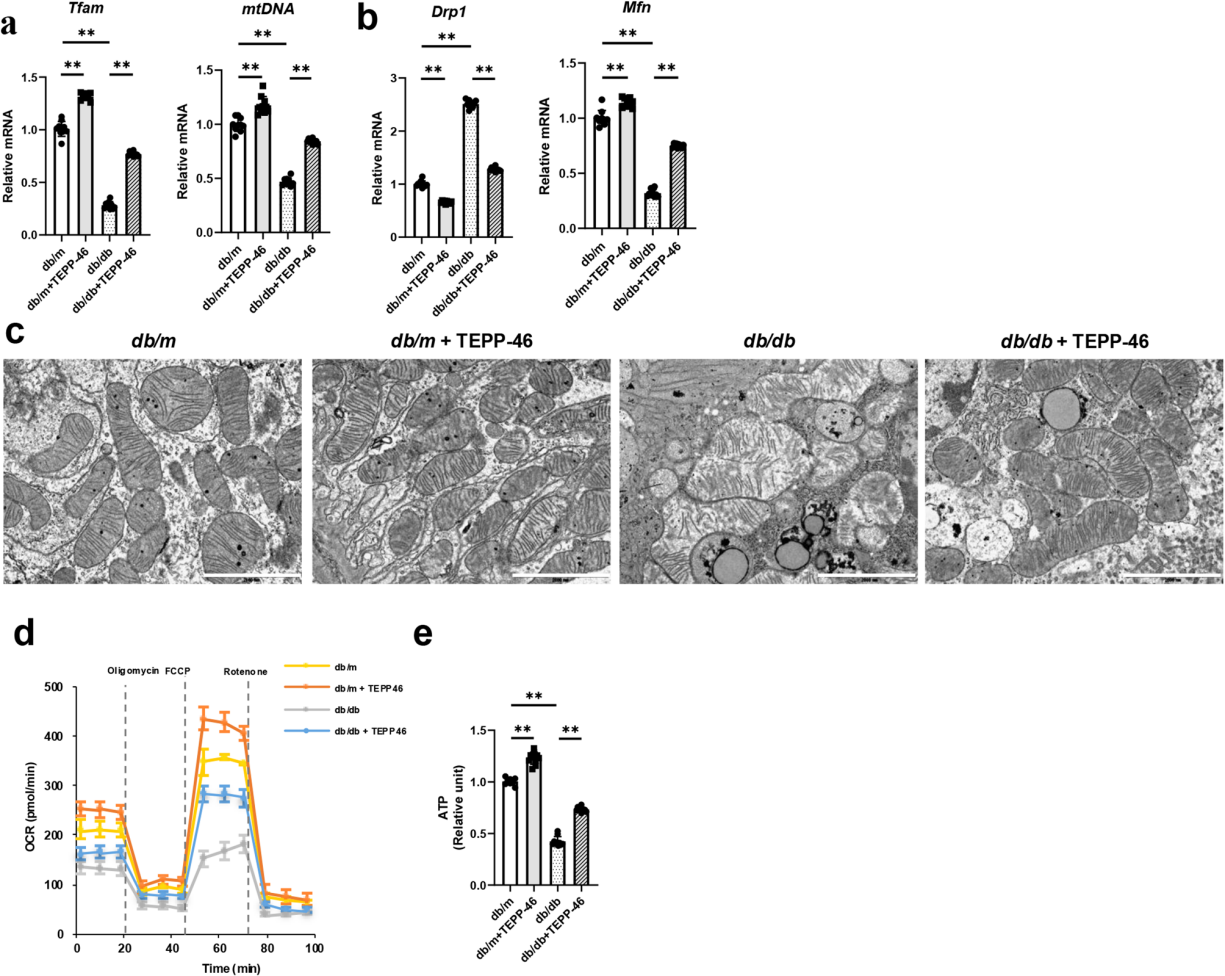
PKM2 activation by TEPP-46 treatment restores altered glycolysis, PGC-1α expression, and fatty acid oxidation in *db/db* mice. Both *db/db* and *db/m* mice were treated with TEPP-46 (10 mg/kg) or dimethyl sulfoxide (20 μL) via intraperitoneal injection for 6 weeks. **a **The PKM2 tetramer to dimer/monomer ratios were lower in *db/db* mice in a disuccinimidyl suberate-mediated cross-linking study. **b-d** The mRNA expression levels of genes of the glycolysis pathway were altered, and **e** concentrations of L-lactate were increased in *db/db* mice. **f** The mRNA expression levels of *Ppargc1a* and the corresponding protein levels of PGC-1α were reduced in *db/db* mice, which were restored upon TEPP-46 treatment. **g** The mRNA and protein expression levels of CPT1 and ACOX1, markers for fatty acid oxidation, were reduced in *db/db* mice and were increased upon TEPP-46 treatment. **h** The acetyl-CoA levels measured using a colorimetric assay were decreased in *db/db* mice, and the decreased levels were restored by TEPP-46 treatment. For all groups, data are presented as mean ± SD (*n* = 10 per group). ns, *P* ≥0.05; *, *P* <0.05; **, *P* <0.01

The mRNA expression levels of *Tfam* and *mtDNA*, indicative of mitochondrial mass, were reduced in *db/db* mice. These decreased levels were significantly increased by TEPP-46 treatment (Fig. 3a). Additionally, the mRNA level of *Mfn*, a mitochondrial outer membrane fusion-related gene, exhibited a notable decrease, whereas that of *Drp1*, a mitochondrial fission-related gene, increased in diabetic mice. These changes were reversed by TEPP-46 treatment (Fig. 3b). Electron microscopy also showed fragmented mitochondria and disrupted cristae in RTECs from *db/db* mice, and these abnormalities were significantly improved by TEPP-46 treatment (Fig. 3c). This defect in mitochondrial metabolism was confirmed by measuring the oxygen consumption rate (OCR). Oxidative phosphorylation was reduced in *db/db* mice compared with *db/m* mice as indicated by decreases in basal respiration, spare respiratory capacity, and ATP production in RTECs from diabetic mice. These alterations were significantly improved by TEPP-46 (Fig. 3d). A colorimetric assay demonstrated decreased energy utilization in *db/db* mice, as evidenced by reduced ATP levels. TEPP-46 treatment significantly increased the ATP levels (Fig. 3e). These findings were consistent with in vitro results (Fig. S2). TEPP-46 treatment attenuated the decreased immunofluorescence intensity of cytochrome c oxidase subunit 4 (COX IV) in RTECs stimulated with HG (Fig. S2n). Suppressed glycolysis, as indicated by reduced extracellular acidification rate (ECAR), was observed in RTECs stimulated with HG, and this effect was partially reversed by TEPP-46 treatment (Fig. S2p). In aggregates, in mice and RTECs under diabetic conditions, there were significant defects in mitochondrial biogenesis, metabolism, and function, which were alleviated by PKM2 activation.

### PKM2 activation via TEPP-46 treatment attenuates kidney fibrosis and apoptosis

**Fig. 4:**
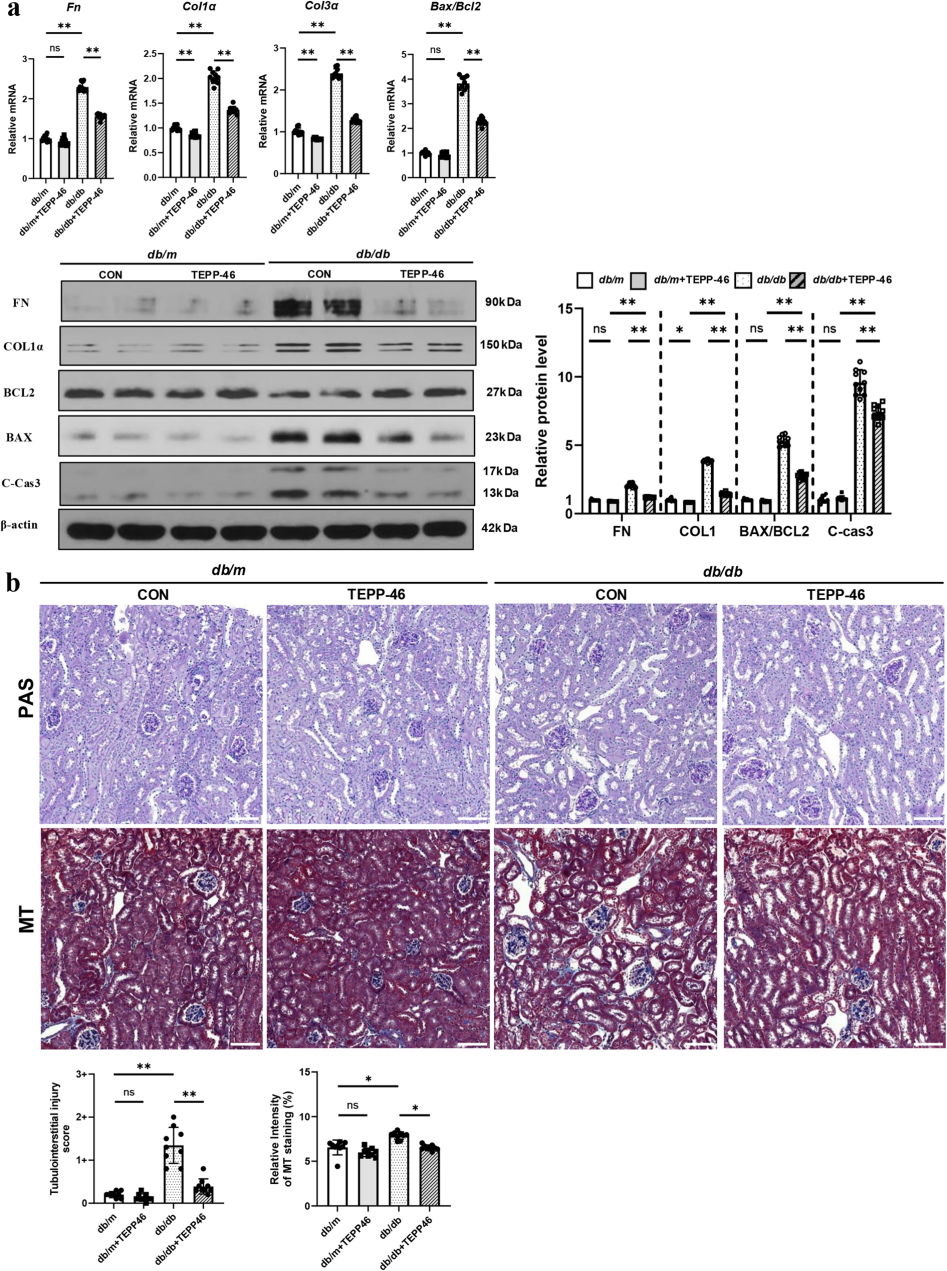
PKM2 activation by TEPP-46 treatment restores kidney fibrosis and cell death in *db/db* mice. Both *db/db* and *db/m* mice were treated with TEPP-46 (10 mg/kg) or dimethyl sulfoxide (20 μL) via intraperitoneal injection for 6 weeks. **a** The mRNA and protein expression levels of pro-fibrotic markers and apoptotic cell death markers were attenuated in *db/db* mice with TEPP-46 treatment. Histological examinations by **b** Periodic acid–Schiff and Masson’s trichrome staining showed the attenuated fibrotic changes in *db/db* mice with TEPP-46 treatment. For all groups, data are presented as mean ± SD (*n* = 10 per group). ns, *P* ≥ 0.05; *, *P* < 0.05; **, *P* < 0.01

The abnormal energy metabolism and mitochondrial dysfunction resulted in kidney fibrosis and tubular cell death. The mRNA and protein levels of fibrotic markers including fibronectin (*Fn*), collagen type I alpha (*Col1a*), collagen type III alpha (*Col3a*), and apoptosis index of BAX/BCL-2 ratio increased in *db/db* mice (Fig. 4a). These changes were similar in HG-stimulated RTECs (Fig. S7). Histological examination revealed tubular degeneration, widening of the interstitium, and interstitial fibrosis in *db/db* mice and Masson’s trichrome staining confirmed the increased fibrotic area (Fig. 4b). However, TEPP-46 treatment reduced the expression levels of fibrotic markers and apoptosis indexes and attenuated fibrotic changes.

### PKM2 activation restores altered energy metabolism and mitochondrial dysfunction in *Hif1a*-overexpressing RTECs

**Fig. 5:**
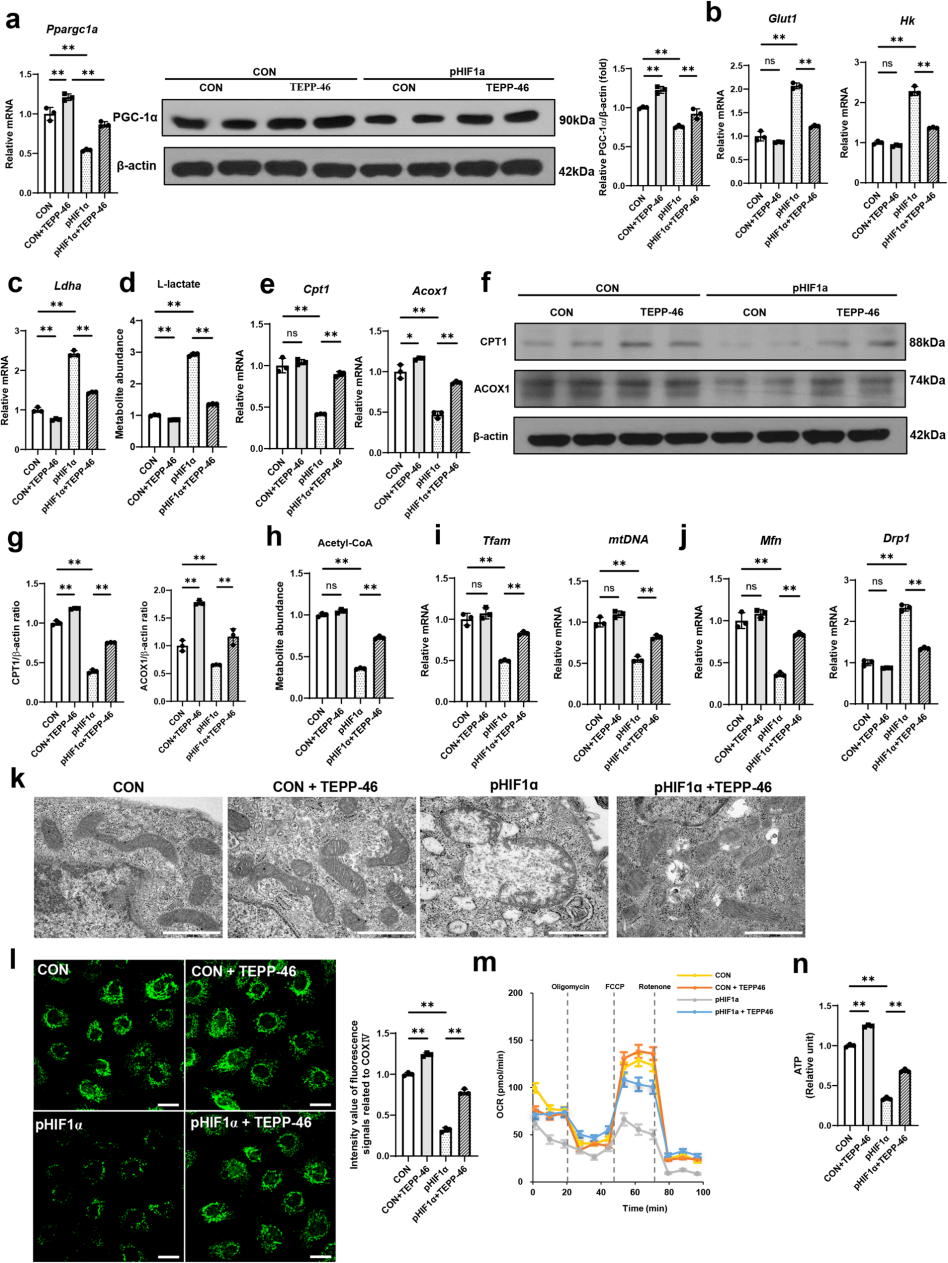
PKM2 activation by TEPP-46 treatment restores PGC-1α expression, and reverses alterations in energy metabolism and mitochondrial dysfunction in *Hif1ɑ*-overexpressing renal tubular epithelial cells (RTECs). Primary RTECs were transfected with the *Hif1a* plasmid (pHIF1α) or vector plasmid and 10 µM TEPP-46 for 48 h. **a** The mRNA expression levels of *Ppargc1a* and the corresponding protein levels of PGC-1α were reduced in pHIF1α-transfected RTECs, and this reduction was reversed by TEPP-46 treatment. **b**, **c** The mRNA expression of genes of the glycolysis pathway were altered; this alteration was reversed by co-treatment with TEPP-46. **d** The concentration of L-lactate was increased in *Hif1a*-overexpressing RTECs, and this increase was reversed by co-treatment with TEPP-46. **e**-**g** The mRNA expression and protein levels of CPT1 and ACOX1, markers of fatty acid oxidation, were reduced in pHIF1α-transfected RTECs, and this reduction was attenuated by TEPP-46 treatment. **h** The acetyl-CoA levels measured by the colorimetric assay were decreased in RTECs with *Hif1a*. **i** The decreased mRNA expression levels of mitochondrial transcripts in pHIF1α-transfected RTECs transfected with pHIF1α were restored by TEPP-46 treatment. **j** The mRNA transcript analysis of *Mfn* and *Drp1* showed a shift in mitochondrial dynamics toward mitochondrial fragmentation in *Hif1a*-overexpressing RTECs. These alterations were diminished upon TEPP-46 treatment. **k** Electron microscopic images of kidney mitochondria in pHIF1α-transfected RTECs transfected with pHIF1α showed a shift in mitochondrial dynamics toward fission and disruption of mitochondrial integrity. These alterations were attenuated by TEPP-46 treatment (scale bar = 2000 nm). **l** Immunofluorescence staining for cytochrome c oxidase subunit 4 (COX IV). COX IV expression was decreased in *Hif1a*-overexpressing RTECs, whereas TEPP-46 treatment attenuated this alteration (scale bar = 200 μm). **m** Oxygen consumption rates (OCRs) in RTECs. *Hif1a*-overexpressing RTECs showed significant reductions in basal OCRs, spare respiratory capacity, proton leak, and ATP production compared with controls, and these reductions were attenuated by co-treatment with TEPP-46. **n** ATP assays of RTECs measured by the colorimetric assay. *Hif1a*-overexpressing RTECs exhibited a low ATP concentration, and this result was attenuated by TEPP-46 treatment. For all groups, data are presented as mean ± SD (*n* = 3 per group). ns, *P* ≥ 0.05; *, *P* < 0.05; **, *P* < 0.01; CON, control

Since HIF-1α played an important role in the regulation of PGC-1α, a master regulator of mitochondrial biogenesis, we examined whether mitochondrial metabolism and function were hampered by HIF-1α, and whether HIF-1α-induced alterations could be recovered by PKM2 activation. As expected, in *Hif1a*-overexpressing RTECs, the mRNA and protein levels of PGC-1α were significantly reduced (Fig. 5a). Additionally, *Hif1a* overexpression resulted in aberrant glycolysis and suppression of the FAO pathway (Fig. 5b-h). Accordingly, there was a significant accumulation of L-lactate, whereas acetyl-CoA levels declined following *Hif1a* overexpression (Fig. 5d). Furthermore, *Hif1a* overexpression reduced mitochondrial mass and perturbed mitochondrial dynamics, evident by decreased *Mfn* expression and increased *Drp1* expression (Fig. 5i and j). Electron microscopy showed mitochondrial fragmentation and disrupted integrity upon *Hif1a* overexpression (Fig. 5k). The COX IV signal intensities decreased upon *Hif1a* overexpression (Fig. 5l). In a mitochondrial functional assay with the Seahorse analyzer, mitochondrial respiration measured by OCR and ATP production was reduced upon *Hif1a* overexpression (Fig. 5m and n). These mitochondrial abnormalities caused by HIF-1α led to increased expression levels of profibrotic markers and apoptotic cell death (Fig. S8). These alterations were reversed by PKM2 activation with TEPP-46. These findings also confirmed the interaction between HIF-1α and PGC-1α and that this relationship was dependent on PKM2.

### Silencing *Hif1a* reverses altered energy metabolism and mitochondrial dysfunction in HG-treated RTECs

**Fig. 6:**
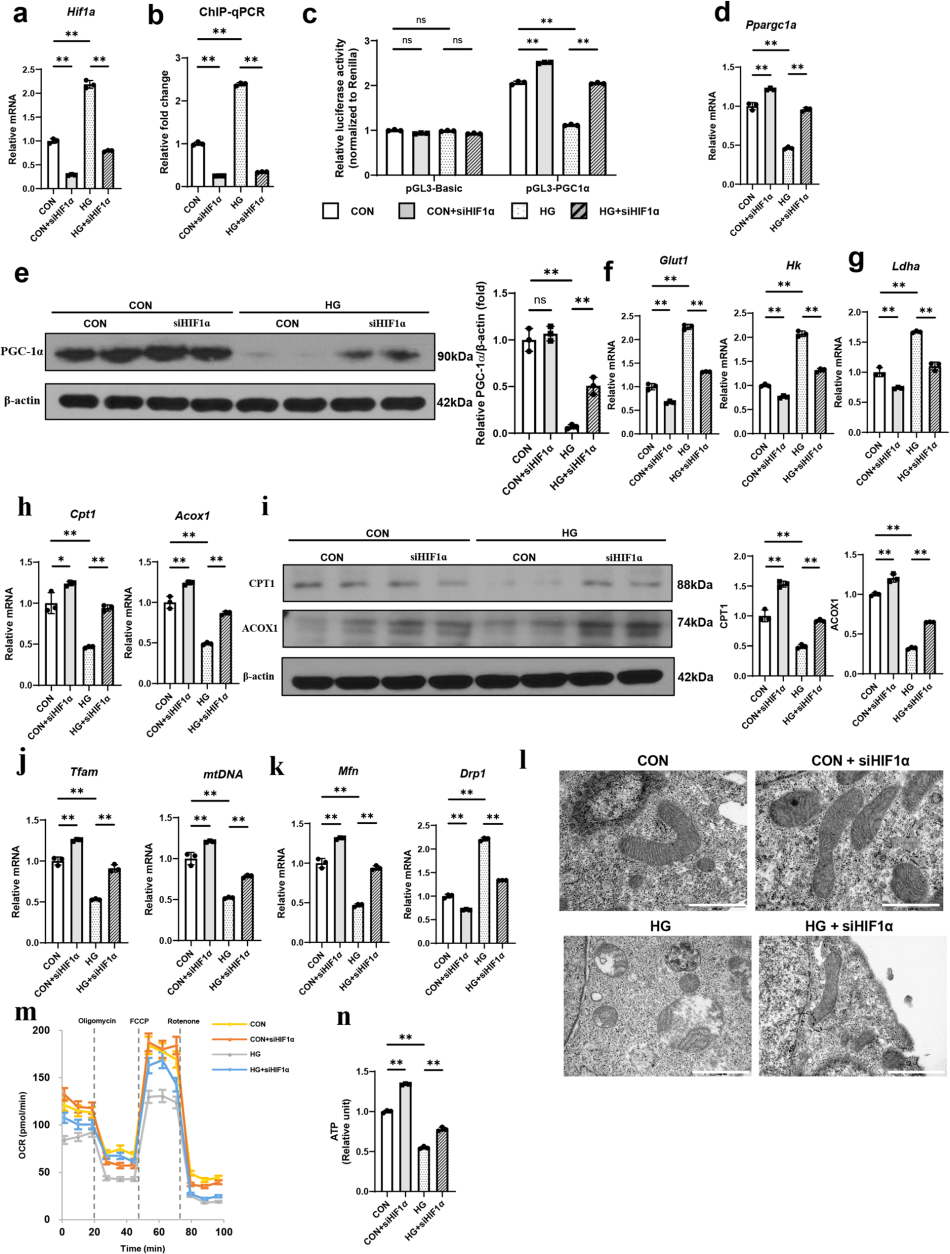
Silencing *Hif1a* alleviates the decreased activity of PGC-1α in HG-treated renal tubular epithelial cells (RTECs). Primary RTECs were transfected with *Hif1a* small interfering RNA (siHIF1α) plasmid or vector plasmid and 40 mM glucose (HG) for 48 h. **a** The mRNA expression levels of *Hif1a* decreased. **b** In the chromatin immunoprecipitation (ChIP) assay, transfection of RTECs with siHIF1α showed a decreased the enrichment of the hypoxia-responsive element (HRE) binding region in *Ppargc1a* compared with controls. **c** A luciferase reporter activity assay using RTECs from primary culture after co-transfection of pGL3-PGC-1α and siHIF1α plasmids. *Ppargc1a* promoter-driven luciferase reporter activity was significantly higher in siHIF1α plasmid-transfected RTECs. *Hif1a* silencing also reversed HG-induced alterations. **d** The mRNA expression levels of *Ppargc1a* and **e** the corresponding protein levels of PGC-1α were reduced in HG-treated RTECs, and this reduction was reversed by siHIF1α plasmid transfection. **f**-**i** Protein expression levels of genes of the glycolysis pathway and fatty acid oxidation were dysregulated. These changes were reversed by transfection of the siHIF1α plasmid. **j** The decreased mRNA expression levels of mitochondrial transcripts in HG-treated RTECs were restored by *Hif1a* silencing. **k** The mRNA transcript analysis of *Mfn* and *Drp1* showed a shift in mitochondrial dynamics toward mitochondrial fragmentation in HG-treated RTECs. **l** Electron microscopic images of kidney mitochondria in RTECs with HG showed mitochondrial fragmentation and disrupted mitochondrial cristae. These alterations were attenuated by transfection with the siHIF1α plasmid (scale bar = 2000 nm). **m** Compared with the controls, *Hif1a*-deficient RTECs exhibited significant increases in basal oxygen consumption rates (OCRs), spare respiratory capacity, proton leak, and ATP production. **n** *Hif1a* silencing significantly restored ATP levels. For all groups, data are presented as mean ± SD (*n* = 3 per group). ns, *P* ≥ 0.05; *, *P* < 0.05; **, *P* < 0.01; CON, control

To substantiate our findings, we suppressed the *Hif1a* gene expression in HG-treated RTECs. *Hif1a* was effectively silenced by a siRNA against *Hif1a* (siHIF1α) (Fig. 6a). In the ChIP assay, *Hif1a* suppression significantly decreased the enrichment of the HRE binding region in the *Ppargc1a* promoter in HG-treated RTECs (Fig. 6b). In addition, *Ppargc1a*-luciferase reporter activity increased upon *Hif1a* silencing in HG-treated RTECs (Fig. 6c). These findings confirmed the regulatory action of HIF-1α on PGC-1α. Next, we examined metabolic changes induced upon *Hif1a* silencing. The mRNA expression of *Ppargc1a* was restored upon *Hif1a* silencing in HG-treated RTECs (Fig. 6d and e). Additionally, *Hif1a* silencing improved aberrant glycolysis and suppressed FAO in these cells (Fig. 6f and i). Moreover, L-lactate was less accumulated, while acetyl-CoA levels increased upon *Hif1a* silencing (Fig. S9a and S9b). *Hif1a* silencing also restored mitochondrial mass and improved altered mitochondrial dynamics and mitochondrial fragmentation (Fig. 6j and l and Supplemental Fig. S9c). Accordingly, mitochondrial respiration measured by OCR and ATP production increased upon *Hif1a* silencing (Fig. 6m and n). These improvements led to decreased expression of profibrotic markers and apoptotic cell death (Fig. S10).

## Discussion

**Fig. 7:**
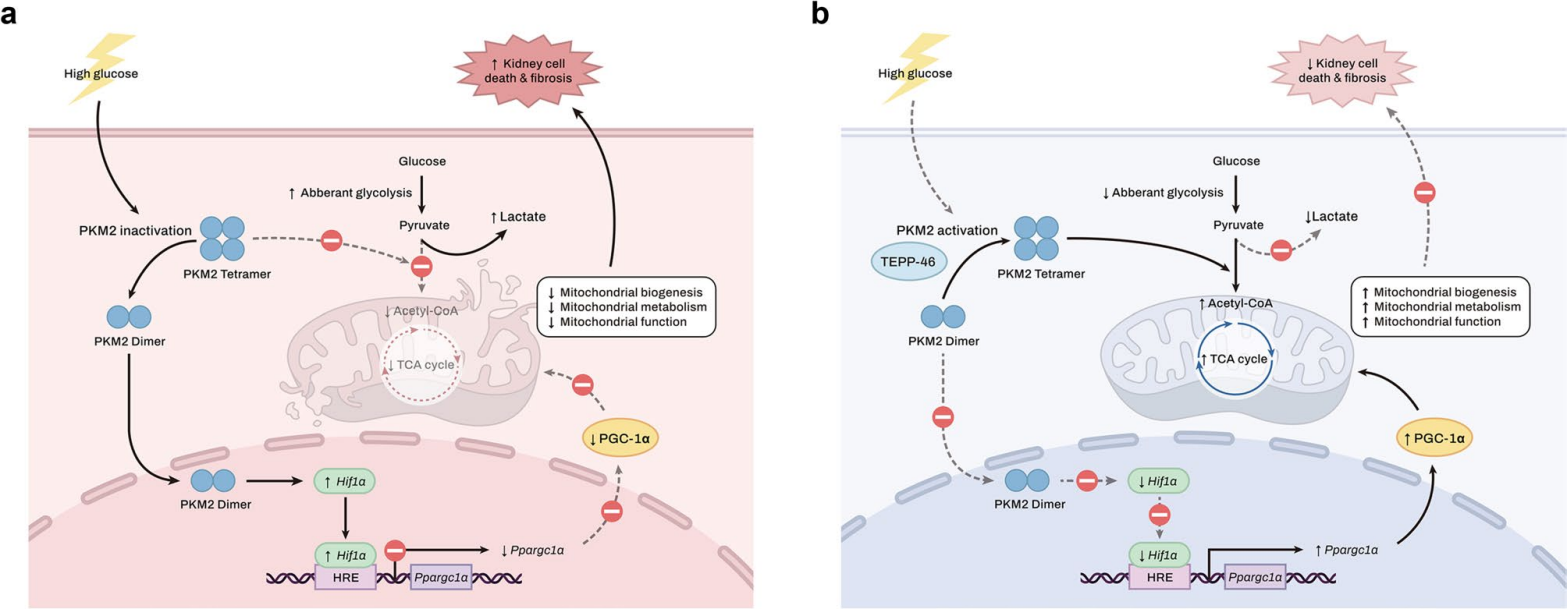
Schematic summary. **a** Under high-glucose conditions, PKM2 inactivation induces mitochondrial dysfunction via HIF-1α-mediated PGC-1α suppression and aberrant glycolysis, which leads to renal tubular cell death and fibrosis. **b** PKM2 activation by TEPP-46 maintains mitochondrial function and energy metabolism, which protects kidney cells from apoptosis and fibrosis

The present study sought to clarify the relationship between PKM2, a glycolytic sensor, and PGC-1α, a key regulator of mitochondrial biogenesis, in a DKD model. We demonstrated that PKM2 activation alleviated the aberrant glycolytic flux, decreased PGC-1α expression, and impaired FAO and mitochondrial metabolism and function in HG-treated RTECs and mice with diabetes. These improvements by PKM2 activation resulted in attenuated cell death and fibrosis. We also found that HIF-1α, a metabolic regulator, was suppressed by PKM2 activation. Notably, HIF-1α directly bound to the regulatory region of the *Ppargc1a* promoter, and this interaction was inversely dependent on PKM2 activation. Thus, enhanced PKM2 activity improved mitochondrial metabolism and function partly through the modulation of the interaction between HIF-1α and PGC-1α. A schematic figure summarizing the mechanisms by which PKM2 activation provides the protective effects against DKD is presented in Fig. 7.

Recent studies demonstrated that prolonged exposure to hyperglycemia induced glycolysis and reduced mitochondrial function in diabetes. Sas et al. reported significantly increased glycolytic intermediates and enzyme levels in glomerular-depleted kidney cortex and suppressed mitochondrial function in *db/db* mice compared with control mice (Sas et al. 2016). Additionally, there was an uncoupling of the electron transport chain in the mitochondria of *db/db* mice. In a human study, TCA cycle-related metabolites and PGC-1α expression significantly decreased in patients with DKD compared with healthy controls (Sharma et al. 2013). Similarly, in our study, kidney tissue from *db/db* mice and HG-treated RTECs exhibited increased *Glut1*, *Hk*, *Ldha*, and *Hif1a* expression and decreased *Pkm2* expression and PKM2 activity. Additionally, lactate accumulation and decreased acetyl-CoA levels were also observed. This aberrant glycolytic flux led to impaired mitochondrial respiration and mitochondrial ATP production. Interestingly, we observed a suppressed oxidative phosphorylation and glycolytic response under hyperglycemic conditions, which was restored by TEPP-46 treatment. While acute glucose administration during the ECAR assay can elicit robust glycolytic responses in naïve cells, our goal was to capture the metabolic phenotype of chronically stressed diabetic cells. The resulting blunted ECAR response under HG — and its rescue by TEPP-46 — likely reflects restoration from a pathophysiologic “metabolic shutdown,” rather than an artifact of glucose exposure timing. Thus, although HG is often assumed to enhance glycolysis, prolonged hyperglycemia can paradoxically suppress glycolytic capacity due to mitochondrial dysfunction, redox imbalance, or feedback inhibition of key enzymes. Our findings are consistent with previous reports in podocytes under diabetic stress, where HG reduced ECAR and PKM2 activation rescued glycolytic flux (Fu et al. 2022; Qi et al. 2017).

Given the significant metabolic shift to glycolysis and mitochondrial damage in the development of DKD, it is meaningful to explore the role of the aberrant glycolytic flux in mitochondrial dysfunction in DKD. This critical issue was recently examined by (Qi et al. 2017). Using unbiased proteomic analysis, they specifically focused on PKM2 because its expression was significantly upregulated in individuals who were protected from DKD compared with those who were not. PKM2-specific activation with TEPP-46 reduced toxic metabolites induced by hyperglycemia and restored the altered mitochondrial metabolism partially by improving glycolytic flux and preserving PGC-1α activity in mice with diabetes. Notably, the mRNA expression of *Ppargc1a* was lower in the kidney of mice with streptozotocin-induced diabetes, which was restored by TEPP-46 treatment. Supporting these findings, TEPP-46 increased PGC-1α expression in macrophages and induced tolerance to LPS in an LPS-induced inflammation model (Yi et al. 2020). Because a mechanistic link between PKM2 and PGC-1α was unclear, herein, we further investigated how PKM2 regulates PGC-1α in the context of mitochondrial metabolism.

Previous studies on cancer metabolism and inflammation have reported that the PKM2 dimer translocates to the nucleus and promotes HIF-1α-mediated transactivation (Palsson-McDermott et al. 2015; Wang et al. 2014). This HIF-1α-dependent transcription factor increases the expression of glycolysis-related genes while simultaneously decreasing mitochondrial function (Fukuda et al. 2007; Maxwell et al. 1997; Palsson-McDermott et al. 2015; Papandreou et al. 2006). Notably, this action is exclusively mediated by PKM2, and not PKM1, independent of the catalytic activity of PKM2 as a glycolytic enzyme (Luo et al. 2011). Moreover, a recent study reported that the PKM2 tetramer induced by TEPP-46 suppressed HIF-1α expression and decreased the expression of epithelial-to-mesenchymal transition markers (Liu et al. 2021). Similarly, we observed that the transcript level of *Hif1ɑ* increased in HG-treated RTECs and kidney of *db/db* mice, and this increased *Hif1ɑ* expression was attenuated by PKM2 activation with TEPP-46. Interestingly, there is evidence that PGC-1α is suppressed by HIF-1α in renal cancer cells and hepatocytes (LaGory et al. 2015; Liu et al. 2014; Tsukada et al. 2013). This evidence led us to hypothesize that PKM2 dimer-induced activation of *Hif1ɑ* may be a key mechanism for suppressing *Ppargc1* expression. Using ChIP and luciferase reporter assays, we demonstrated that HIF-1α directly regulated *Pargc1a* transcription. Overexpression of *Hif1a i*n RTECs by HG and *Hif1a* plasmid led to a notable increase in the enrichment of the HRE binding region within the *Ppargc1a* promoter, consequently resulting in decreased PGC-1α expression. Conversely, TEPP-46-induced PKM2 activation and *Hif1ɑ* silencing reduced HIF-1α binding to the *Ppargc1a* promoter. In RTECs with enhanced HIF-1α induction, concomitant defects in mitochondrial morphology and function were observed, which were reversed by TEPP-46–induced PKM2 activation and *Hif1α* silencing. These findings suggest that PKM2 activation by TEPP-46 downregulates HIF-1 α expression even in the context of forced *Hif1a* overexpression, thereby relieving PGC-1α suppression. Therefore, based on these findings, we postulate that direct interactions between HIF-1α and PGC-1α are regulated by PKM2, and modulating the effects of PKM2 on these key metabolic regulators may protect against diabetic kidney injury in RTECs. Supporting our findings, upregulated PKM2 expression in renal tissues from long standing diabetic individuals protected from DKD and down regulated PGC-1α and impaired TCA cycle activity were observed in previous studies (Qi et al. 2017; Sharma et al. 2013). These clinical findings are consistent with our preclinical observations that PKM2 activation via TEPP-46 restores PGC-1α levels, rescues mitochondrial dysfunction, and mitigates kidney injury. While clinical trials directly targeting PKM2 in diabetic patients are not yet available, our results support the translational potential of PKM2 activators in human DKD. Although, the PKM2–HIF-1α–PGC-1α axis appears central, our data suggest that PKM2 also exerts effects independent of HIF-1α. The observation that TEPP-46 partially restored *Ppargc1a* expression under *Hif1a* silencing highlights the presence of additional regulatory pathways that merit further investigation.

Our study also highlights the importance of PGC-1α in preserving mitochondrial metabolism and function. Fine-tuning the expression of PGC-1α is essential for maintaining mitochondrial homeostasis, which is determined by the balance between mitochondrial biogenesis and clearance. PGC-1α is a master regulator of this process, which promotes mtDNA duplication and coordinates the gene expression of key components of mitochondrial biogenesis to produce healthy organelles that replace defective mitochondria (Campbell et al. 2012). A notable discovery from our study is the pivotal role of HIF-1α in finely regulating PGC-1α transcriptional activity. There are numerous factors that control PGC-1α expression (Miller et al. 2019). Previous reports suggest that HIF-1α may indirectly influence PGC-1α activity by suppressing sirtuin 2 (SIRT2)-mediated deacetylation in dietary obesity mouse models (Krishnan et al. 2012). In parallel, AMP-activated protein kinase (AMPK) activation promotes PGC-1α transcription and mitochondrial biogenesis (Bergeron et al. 2001; Zong et al. 2002), whereas excessive mammalian target of rapamycin (mTOR) activation has been associated with suppression of mitochondrial function (Cunningham et al. 2007). Future studies are warranted to explore how PKM2 activation may interface with other indirect pathways-including AMPK/mTOR signaling networks and SIRT2-to fine-tune energy metabolism in DKD. Notably, to our knowledge, no studies have clearly delineated the direct regulatory action of HIF-1α on PGC-1α. Conversely, there may be an inverse relationship between these two factors given that disruption of mitochondria due to PGC-1α deficiency can result in an ineffective TCA cycle, leading to accumulation of TCA intermediates such as fumarate and succinate (Hasegawa and Inagi 2021; Zhang et al. 2018). These two intermediate products can inhibit the prolyl hydroxylase domain, which in turn can activate HIF-1α (Isaacs et al. 2005; Tannahill et al. 2013; You et al. 2016). These complex interactions should be further studied in the future.

This study has several limitations. We employed a high-glucose concentration (40 mM) in vitro to mimic hyperglycemic stress. Although this concentration is commonly employed in diabetic models, it exceeds the physiological glucose levels typically observed in diabetic models. To address this, we repeated key experiments assessing changes in transcript levels related to glycolysis, fatty acid oxidation and mitochondrial mass using 30 mM glucose, a level that more closely reflects the upper pathological range in diabetic mice and observed comparable results. However, we cannot fully exclude the possibility that some of the observed metabolic and mitochondrial alterations may reflect exaggerated responses to supraphysiological glucose levels. Future studies employing chronic exposure to physiologically relevant hyperglycemia could further validate and strengthen our findings. Although our study specifically examined PKM2 activation and mitochondrial preservation in RTECs, mitochondrial dysfunction in podocytes is also crucial in DKD progression (Forbes et al. 2018). Future studies will be necessary to determine whether the protective effects of PKM2 activation on mitochondrial metabolism extend to podocytes and whether the PKM2-HIF-1α-PGC-1α regulatory axis similarly operates in glomerular cells.

## Conclusion

This study showed that PKM2 activation mitigated compromised mitochondrial metabolism and function in diabetic mice and HG-treated RTECs. Furthermore, we also demonstrated that HIF-1α directly regulated PGC-1α activity in a PKM2-dependent manner. Thus, the regulatory actions of PKM2 on the interaction between HIF-1α and PGC-1α play an important role in DKD.

## Supplementary Information

Supplementary Material 1.

## Data Availability

No datasets were generated or analysed during the current study.

## Abbreviations

acetyl-CoA: Acetyl coenzyme A
ChIP: Chromatin immunoprecipitation
COX IV: Cytochrome c oxidase subunit 4
DSS: Disuccinimidyl suberate
DKD: Diabetic kidney disease
KFRT: Kidney failure with replacement therapy
FAO: Fatty acid oxidation
HG: High glucose
HRE: Hypoxia-responsive element
OCR: Oxygen consumption rate
PBS: Phosphate-buffered saline
PGC-1α: Peroxisome proliferator-activated receptor-γ coactivator-1α
PKM2: Pyruvate kinase isoform M2
RTECs: Renal tubular epithelial cells
siRNA: Small interfering RNA
