# Supplementary material for: Pyruvate kinase M2 activation maintains mitochondrial metabolism by regulating the interaction between HIF-1α and PGC-1α in diabetic kidney disease
Source: Mol Med. 2025 Jul 25;31:266. doi: 10.1186/s10020-025-01320-4 (PMC12291527; doi:10.1186/s10020-025-01320-4)
Supplement: Supplementary file 1 — Supplementary Material 1. [file 10020_2025_1320_MOESM1_ESM.docx]

**SUPPLEMENTARY INFORMATION**

**Pyruvate kinase M2 activation maintains mitochondrial metabolism by regulating the interaction between HIF-1α and PGC-1α in diabetic kidney disease**

**Corresponding Author:** Seung Hyeok Han, M.D., Ph.D.

Department of Internal Medicine, College of Medicine, Institute of Kidney Disease Research, Yonsei University, 50-1 Yonsei-ro, Seodaemun-gu, Seoul, Korea, 03722

Phone: 82-2-2228-1984/Fax: 82-2-393-6884/E-mail: [hansh@yuhs.ac](mailto:hansh@yuhs.ac)

**Table of contents**

**Supplementary complete materials and methods**

**Supplementary Table S1.** Primer sequences

**Supplementary Table S2.** Body weight, kidney weight, and biochemical parameters

**Supplementary Figure S1.** Blood glucose during the experiment period.

**Supplementary Figure S2.** PKM2 activation by TEPP-46 treatment improves aberrant glycolytic flux and decreases PGC-1α expression and fatty acid oxidation in high glucose-treated renal tubular epithelial cells.

**Supplementary Figure S3.** High glucose, not osmotic pressure, induces decreased PKM2 activation and PGC-1α expression in renal tubular epithelial cells.

**Supplementary Figure S4.** PKM2 activation by TEPP-46 treatment improves aberrant glycolytic flux, fatty acid oxidation, and mitochondrial dysfunction in 30 mM glucose-treated renal tubular epithelial cells.

**Supplementary Figure S5.** PKM2 regulates HIF-1α expression and PGC-1α activity in renal tubular epithelial cells.

**Supplementary Figure S6.** PKM2 regulates PGC-1α activity via both HIF-1α–dependent and –independent mechanisms.

**Supplementary Figure S7.** PKM2 activation by TEPP-46 treatment restores kidney fibrosis and apoptosis in high glucose-treated renal tubular epithelial cells.

**Supplementary Figure S8.** PKM2 activation by TEPP-46 treatment restores kidney fibrosis and apoptosis in HIF-1α-overexpressing renal tubular epithelial cells.

**Supplementary Figure S9.** Silencing *Hif1a* reverses altered energy metabolism and mitochondrial dysfunction in high glucose-treated renal tubular epithelial cells.

**Supplementary Figure S10.** Silencing *Hif1a* decreases the expression of fibrotic markers and apoptosis in high glucose (HG)-treated renal tubular epithelial cells.

**Supplementary complete materials and methods**

1. **Primary culture of renal tubular epithelial cells (RTECs) and transfection**

Mouse kidney RTECs were isolated from Ten male C57BL/6 mice. Dissected kidneys were minced into pieces of approximately 1 mm^3^ per 1 mL ice-cold Dulbecco’s phosphate-buffered saline (DPBS) (Gibco, Thermo Fisher Scientific, Waltham, MA, USA). The fragments were transferred to a 1 mg/mL collagenase solution in DPBS (Sigma-Aldrich, St. Louis, MO, USA) and digested for 30 min at 37°C. Then, the supernatants were sieved through a 100-μm nylon mesh followed by centrifugation for 10 min at 3000 rpm. Sterile red blood cell lysis buffer (8.26 g NH_4_Cl, 1 g KHCO_3_, 0.037 g EDTA per 1 L double-distilled H_2_O) was applied to a pellet and incubated on ice for 10 min, followed by centrifugation for 10 min at 3000 rpm. The pellet was washed twice with DPBS and seeded in 10-cm dishes. RTECs were cultured in DMEM media (Thermo Fisher Scientific, Waltham, MA, USA), supplemented with 10% fetal bovine serum (FBS) (Thermo Fisher Scientific, Waltham, MA, USA), 100 units/mL penicillin G (Sigma-Aldrich, St. Louis, MO, USA), 2.5 μg/mL amphotericin B (Sigma-Aldrich, St. Louis, MO, USA), and 20 ng/mL epidermal growth factor (Sigma-Aldrich, St. Louis, MO, USA).

At a confluency of approximately 70%, the RTECs were FBS-restricted for 24 h, and the medium was replaced with 1% FBS DMEM medium for the control group and with D-glucose (40 mM) to induce a high-glucose (HG) environment similar to that observed in diabetes. RTECs were harvested 48 h after medium changes. In this study, we used TEPP-46, a small-molecule PKM2 activator (Selleck Chemicals, Houston, TX, USA), to induce PKM2 activation. This molecule stabilizes PKM2 subunit interactions and increases PK activity, resulting in the formation of the PKM2 tetramer (D Anastasiou et al., 2012). We treated the cells with 10 μM TEPP-46 in all experiments based on a previous study.(W Qi et al., 2017). In *Hif1a* and *Pkm2* suppression experiments, RTECs were transfected with a mouse *Hif1a* small interfering RNA plasmid (siRNA), *Pkm2* siRNA (GE Dharmacon, Buckinghamshire, UK), or both using Lipofectamine RNAiMAX reagents (Invitrogen, Carlsbad, CA, USA), and media were changed to serum-free media after 6 h of transfection. For *Hif1a* overexpression, cells were transfected with mouse *Hif1a* plasmid (1 μg) or vector plasmid (Addgene, Cambridge, MA, USA) using Lipofectamine 3000 and Plus reagents (Invitrogen, Carlsbad, CA, USA). The cells were then incubated for an additional 48 h and analyzed.

1. **Animal study and treatment**

For the animal study of type 2 diabetes, we purchased B6.Cg-M+/+Lepr^db^/Lepr^db^ (*db/db*) male mice and their heterozygous littermates B6.Cg-M+/-Lepr^db^/+ (*db/m*) as non-diabetic controls from Jackson Laboratories (Bar Harbor, ME, USA). Animals were randomized to treatment groups to minimize bias. Ten male mice each in the diabetes and control groups were intraperitoneally injected with TEPP-46 (10 mg/kg) or dimethyl sulfoxide, respectively, at 6 weeks of age for 6 weeks. After 6 weeks, the kidneys were extracted under anesthesia induced with Zoletil (10 mg/kg) (Virbac, Carros, France), after which the animals were sacrificed. Kidney samples were immediately frozen in liquid nitrogen and stored at -70°C until use. The blood samples were collected via the left ventricle using a 23-gauge needle (S Parasuraman et al., 2010).

1. **Cross-linking for determination of PKM2 isomers**

To assess the status of PKM2 subunit association, we conducted a disuccinimidyl suberate (DSS)-mediated cross-linking study. RTECs were lysed in PBS with 0.5% Triton X-100 (Sigma-Aldrich, St. Louis, MO, USA) and scraped from culture plates. Cells from kidney tissue samples were lysed in PBS with 0.5% Triton X-100 and a protease inhibitor (Thermo Fisher Scientific, Waltham, MA, USA) for 30 min at 4℃. Fresh DSS (2 mM) (Thermo Fisher Scientific, Waltham, MA, USA) was then added to the samples and incubated for 30 min at 37℃. A quenching solution with 1 M Tris was added to the samples and incubated for 15 min at room temperature (RT). The cross-linked samples were added to 4X NuPAGE LDS sample buffer (Thermo Fisher Scientific, Waltham, MA, USA) and boiled for 5 min at 100℃. The samples were then separated by 4–12% Bis-Tris gradient gel (Thermo Fisher Scientific, Waltham, MA, USA) and transferred to polyvinylidene difluoride membranes. The membranes were incubated with 0.4% paraformaldehyde in PBS for 30 min at RT. PKM2 antibody (Cell Signaling Technology, Danvers, MA, USA) was then added for detection of PKM2 tetramers, dimers, and monomers.

1. **Total RNA extraction**

For RTECs, 700 μL RNAiso reagent (Takara Bio Inc., Otsu, Japan) was added to cell culture dishes, and the cell suspensions were collected and homogenized for 5 min at RT. The whole mouse kidney samples were rapidly frozen using liquid nitrogen and homogenized in 700 μL RNAiso reagent using a mortar and pestle, thrice. Then, 160 µL chloroform was applied into the homogenized samples of kidneys and RTECs. The mixtures were agitated vigorously for 30 s, incubated for 3 min at RT, and centrifuged at 12,000 rpm for 15 min at 4℃. Among the separated layers, samples from the aqueous layer, located at the top of the three layers, were transferred to a fresh tube, precipitated by adding 400 μL isopropanol, and pelleted by centrifugation at 12,000 rpm for 30 min at 4°C. The RNA precipitate was washed with 70% ethanol, air-dried for 2 min, and dissolved in sterile diethyl pyrocarbonate-treated distilled water. The quantity and quality of the extracted RNA were assessed by spectrophotometric measurements at 260- and 280-nm wavelengths.

1. **Reverse transcription**

First-strand complimentary DNA (cDNA) from extracted RNA was synthesized using a cDNA synthesis kit (Takara Bio Inc., Otsu, Japan). Two micrograms of total RNA was reverse transcribed using a 10 μM random hexanucleotide primer, 1 mM dNTP, 8 mM MgCl_2_, 30 mM KCL, 50 mM Tris-HCl at pH 8.5, 0.2 mM dithiothreitol, 25 U RNase inhibitor, and 40 U PrimeScript reverse transcriptase. The mixture was incubated for 10 min at 30℃, and for 1 h at 42℃, followed by incubation for 5 min at 99℃ for the inactivation of the enzyme.

1. **Real-time quantitative polymerase chain reaction**

Quantitative real-time polymerase chain reaction (qPCR) was conducted in a mixture with a total volume of 20 μL, containing 10 μL SYBR Green PCR Master Mix (Applied Biosystems, Foster City, CA, USA), 5 μL reverse-transcribed cDNA, and 5 pM sense and antisense primers. The sequences of primers are presented in Supplementary Table 1. The primer concentrations were optimized from preliminary experiments designed to find the ideal concentrations of each primer. All PCRs started with an initial heating step at 95°C for 9 min and ended with a final extension at 72°C for 7 min, after repetitive cycles. The qPCR was performed under the following repetitive conditions: 35 cycles of denaturation at 94.5°C for 30 min, annealing at 60°C for 30 s, and extension at 72°C for 1 min. Each sample underwent qPCR in triplicate in separate tubes, and a control without cDNA was also subjected to the protocol in parallel with each assay. After qPCR, the temperature was increased from 60 to 95°C at a rate of 2°C per minute to construct a melting curve. The cDNA content of each specimen was determined using a comparative CT method with 2^-ΔΔCT^. The results were obtained as the relative expression normalized to the expression of 18s ribosomal RNA (rRNA) and expressed in arbitrary units. qPCR was performed to compare the transcript levels of genes related to glycolysis (*Glut1*, *Hk*, *Pkm1*, *Pkm2*, *Hif1a*, and *Ldha*), fatty acid oxidation (FAO) (*Cpt* and *Acox1*), *Ppargc1a*, mitochondrial transcripts (*Tfam* and *mtDNA*), mitochondrial dynamics (*Mfn* and *Drp1*), fibrosis (*Fn1*, *Cola1a1*, and *Cola1a3*), and apoptosis (*Bax* and *Bcl-2*). All primers were obtained from Applied Biosystems (Foster City, CA, USA).

1. **Western blot analysis**

The levels of glycolysis-related proteins, the FAO pathway, fibrosis, and apoptosis were compared using western blot analysis. Harvested cultured RTECs and kidneys were lysed in sodium dodecyl sulfate (SDS) sample buffer containing 2% SDS, 10 mM Tris-HCl with a pH of 6.8, and 10% (vol/vol) glycerol. The lysate was centrifuged at 10,000 rpm for 10 min at 4°C, and the supernatant was stored at -70°C until use. Protein concentrations were determined using a Bio-Rad kit (Bio-Rad Laboratories, Inc., Hercules, CA, USA). Laemmli sample buffer was added to 50-μg aliquots of the protein extracts, which were then heated for 5 min at 100°C and electrophoresed in acrylamide-denaturing SDS-polyacrylamide gel. The separated proteins were transferred to a Hybond-ECL membrane (Sigma-Aldrich, St. Louis, MO, USA) with a Hoeffer semidry blotting apparatus (Hoeffer Instruments, San Francisco, CA, USA). Then, the membrane was incubated in blocking buffer A, containing 1 × TBS, 0.1% Tween 20, and 5% skimmed milk for 30 min at RT, and incubated overnight at 4°C with primary antibodies (at a dilution of 1:1,000) against the proteins. The following were used as primary antibodies to evaluate the expression of proteins of interest: PGC-1α (Abcam, Cambridge, MA, USA), carnitine palmitoyltransferase 1 (CPT1) (Novus Biologicals, Littleton, CO, USA), peroxisomal acyl-coenzyme A oxidase 1 (ACOX1) (Abcam, Cambridge, MA, USA), hypoxia-inducible factor 1-alpha (HIF-1α) (Novus Biologicals, Littleton, CO, USA), fibronectin (DAKO, Carpentaria, CA, USA), type I collagen (Southern Biotech, Birmingham, AL, USA), B-cell lymphoma 2 (BCL-2) (Santa Cruz Biotechnology, Santa Cruz, CA, USA), Bcl-2-associated X (BAX) (Santa Cruz Biotechnology, Santa Cruz, CA, USA), cleaved caspase 3 (Cell Signaling Technology, Danvers, MA, USA), and β-actin (Sigma-Aldrich, St. Louis, MO, USA). Horseradish peroxidase-conjugated anti-goat, anti-rabbit, or anti-mouse IgG (all from Santa Cruz Biotechnology, Santa Cruz, CA, USA) were used as secondary antibodies. After repeated washing, the membrane was developed using a chemiluminescence reagent (ECL, Amersham Life Science, Loughborough, UK). ImageJ ver. 1.49 (National Institutes of Health, Bethesda, MD, USA; accessible online at http://rsbweb.nih.gov/ij) was used to quantify western blot band densities. Changes in the optical densities of the bands of the treated groups relative to control tissues and cells were analyzed.

1. **Measurement of lactate concentrations**

The L-lactate concentrations were quantified using a colorimetric assay kit (Abcam, Cambridge, MA, USA). Tissue and cell extractions were performed according to manufacturer instructions, and extracted samples were loaded onto a 96-well plate. After application of the reaction mix, the microplate was incubated per manufacturer instructions and the samples were analyzed using a microplate reader at 450 nm wavelength.

1. **Acetyl-coenzyme A (acetyl-CoA) assay**

Acetyl-CoA levels were assessed using a colorimetric assay kit (Abcam, Cambridge, MA, USA). Tissue and cell extract samples were loaded onto a 96-well plate, after which a CoA quencher was applied to the wells to remove the background-free coenzyme A and succinyl coenzyme A, and the samples were incubated for 5 min at RT. A quencher remover was then added to the wells, and the samples were incubated for 5 min at RT again. After application of the reaction mix, the microplate was incubated for 10 min at 37℃ and samples were analyzed with a microplate reader at a wavelength of 450 nm.

1. **Chromatin immunoprecipitation assay**

A chromatin immunoprecipitation (ChIP) assay was conducted as previously described (G Sun et al., 2010). Briefly, 2 × 10^7^ primary culture cells were cross-linked, washed, and sonicated. The resulting lysates were subjected to immunoprecipitation with antibodies against mouse HIF-1α (Abcam, Cambridge, MA, USA) or control IgG (Santa Cruz Biotechnology, Santa Cruz, CA, USA). Protein A agarose/salmon sperm DNA (Millipore, Temecula, CA, USA) was used to capture the immunoprecipitants. Bound proteins were eluted after washing, and ChIP-enriched DNA was isolated by phenol:chloroform extraction. The eluted ChIP DNA and input control samples were analyzed by qPCR using the following primer pair within the *Ppargc1a* enhancer promoter: the primer sequences were sense 5′-CACGTGTGATGTAGCTGGTGCAG-3′ and anti-sense 5′-CTTGCATTCCATTCCTTACACGT-3′. Data were normalized to the input samples as described elsewhere (G Sun et al., 2010).

1. **Luciferase reporter assay**

The *Ppargc1a* promoter luciferase plasmid (pGL3-PGC-1α) and vector-only plasmid (pGL3-basic) were purchased from Addgene (Cambridge, MA, USA). Primary RTECs were obtained as described above and seeded in six-well plates. On the following day, 100 ng Renilla luciferase-encoding plasmid and 400 ng pGL3-basic or pGL3-PGC-1α plasmid were transfected using Lipofectamine 2000 according to the manufacturer’s instructions. The cells were harvested 24 h after transfection and analyzed with the Dual-Luciferase Reporter Assay System (E1910, Promega, Madison, WI, USA). Luminescence was measured on a CentroXS^3^ LB9601 luminometer (Berthold Technologies, Bad Wildbad, Germany). The luciferase activity of each group was normalized to Renilla luciferase activity, and between-group differences were expressed as relative fold changes.

1. **Measurement of oxygen consumption rate and extracellular acidification rate**

An XF24 Extracellular Flux Analyzer (Seahorse Bioscience, Billerica, MA, USA) was used to measure the oxygen consumption rate (OCR) in the medium directly adjacent to adherent cells. Mouse RTECs were seeded in a XF24 V7 cell culture microplate at 1.0 × 10^4^ cells per well and cultured in DMEM supplemented with 1% FBS. RTECs on XF24 microplates were rinsed once and re-suspended in 500–675 μL XF assay buffer (DMEM with glucose 10 mM, pyruvate 1 mM, and glutamine 2 mM). After baseline measurement, the OCR level was obtained at 5-min intervals after addition of the following at working concentrations: oligomycin (2 μm), a mitochondrial ATP synthetase inhibitor, carbonyl cyanide-p-trifluoromethoxyphenylhydrazone (FCCP) (0.5 μM), a proton gradient uncoupler, and rotenone (0.5 μm), a mitochondrial complex I inhibitor, with antimycin A (0.5 μm), a mitochondrial III inhibitor. The OCR was measured in pmol/min.

For extracellular acidification rate (ECAR) measurement, an XF96 Extracellular Flux Analyzer (Seahorse Bioscience, Billerica, MA, USA) was used. mouse RTECs were seeded at 2.0 × 10^3^ cells per well in XF96Fe microplates and cultured in DMEM supplemented with 1% FBS. Prior to the assay, cells were washed once and incubated in XF assay medium (DMEM containing 10 mM glucose, 1 mM pyruvate, and 2 mM glutamine) in a final volume of 180 μL per well. After baseline measurements, ECAR was recorded following sequential injections of glucose (10 mM), oligomycin (1 μM), a mitochondrial ATP synthetase inhibitor, and 2-deoxyglucose (2-DG, 50 mM), a glycolysis inhibitor.

1. **Transmission electron microscopy**

Standard transmission electron microscopy was used for mitochondrial structure examination. Dissected kidney tissues were washed with precooled PBS at a pH of 7.4, postfixed in a mixture of 2% paraformaldehyde and 2.5% glutaraldehyde overnight, washed, dehydrated, and embedded in an epoxy resin following standard procedures. Ultrastructural images were captured with a JEOL 1011 microscope (JEOL, Tokyo, Japan).

1. **Histological analysis**

Periodic acid–Schiff (PAS) staining and Masson’s trichrome (MT) staining were performed on 10% formalin-fixed, paraffin-embedded kidney sections to evaluate histological features. PAS staining was performed with dissolved paraffin on tissue slides in an incubator at 60°C for more than 30 min. The samples were then sequentially rehydrated with xylene and 100%, 95%, and 90% alcohol. After washing the slides with distilled water and then soaking them in periodic acid for 7 min, Schiff’s solution was added to the slides and incubated for 15 min. Modified Mayer’s hematoxylin counter-staining was performed after dehydration. The PAS-stained tissues were used for semi-quantitative tubulointerstitial injury scoring. Tubular injury was scored on a scale of 0 to 4, based on findings of tubular dilatation, loss of brush border and tubular structure, and tubular atrophy in the cortical region: 0 (normal), 1 (<25%), 2 (25%–50%), 3 (50%–75%), and 4 (>75%) (M Nangaku et al., 1998).

For MT staining, 5-μm-thick sections of paraffin-embedded tissues were deparaffinized, rehydrated in ethyl alcohol, washed in tap water, and re-fixed in Bouin’s solution at 56°C for 1 h. After washing the samples in running tap water for 10 min and staining with Weigert’s iron hematoxylin working solution for 10 min, the slides were stained with Biebrich scarlet-acid fuchsin solution for 15 min and washed in tap water. The sections were differentiated in phosphomolybdic-phosphotungstic acid solution for 15 min, transferred to aniline blue solution, and stained for 10 min. After rinsing briefly in tap water, the sections were differentiated with 1% acetic acid solution for 5 min. The MT staining score was determined based on the staining intensity using a digital image analyzer (ImageJ, National Institutes of Health, Bethesda, MD, USA).

For immunofluorescence staining of cytochrome c oxidase subunit 4 (COX IV), anti-mouse COX IV antibody (Abcam, Cambridge, MA, USA) was used. Primary cultured RTECs were seeded on a chambered slide and stimulated using the aforementioned method. After stimulation, RTECs were fixed in 4% paraformaldehyde for 10 min at RT, blocked in DPBS containing 5% normal goat serum for 60 min, and permeabilized with 0.1% Tween 20 for 10 min at RT. The cells were then incubated in anti-mouse COX IV antibody overnight at 4°C, and anti-mouse Alexa Fluor 488 (Cell Signaling Technology, Danvers, MA, USA) was added, after which the cells were imaged. A semiquantitative staining intensity score was obtained after examining at least five fields in each section under ×400 magnification using a digital image analyzer (ImageJ, National Institutes of Health, Bethesda, MD, USA).

1. **Statistical analysis**

All the experimental data are expressed as mean ± standard error of the mean (SEM) and analyzed using GraphPad Prism 9.4 (GraphPad Software, San Diego, CA, USA). The data were used in the analysis without applying any specific handling for outliers or missing values. One-way analysis of variance (ANOVA) was performed, followed by the Tukey test for multiple comparisons and Student’s t-test for single comparisons. A *P*-value <0.05 was considered to indicate statistical significance.

1. **Supplemental references**

1. Anastasiou D, Yu Y, Israelsen WJ, Jiang JK, Boxer MB, Hong BS, et al. Pyruvate kinase M2 activators promote tetramer formation and suppress tumorigenesis. Nat Chem Biol. 2012;8(10):839-47.

2. Nangaku M, Alpers CE, Pippin J, Shankland SJ, Kurokawa K, Adler S, et al. CD59 protects glomerular endothelial cells from immune-mediated thrombotic microangiopathy in rats. J Am Soc Nephrol. 1998;9(4):590-7.

3. Parasuraman S, Raveendran R, Kesavan R. Blood sample collection in small laboratory animals. J Pharmacol Pharmacother. 2010;1(2):87-93.

4. Qi W, Keenan HA, Li Q, Ishikado A, Kannt A, Sadowski T, et al. Pyruvate kinase M2 activation may protect against the progression of diabetic glomerular pathology and mitochondrial dysfunction. Nat Med. 2017;23(6):753-62.

5. Sun G, Reddy MA, Yuan H, Lanting L, Kato M, Natarajan R. Epigenetic Histone Methylation Modulates Fibrotic Gene Expression. Journal of the American Society of Nephrology. 2010;21(12):2069-80.

**Supplementary Table S1.** Primer sequences

| **Mouse Gene** | **Sequence (5′→3′)** | |
| --- | --- | --- |
|  | **Forward** | **Reverse** |
| *Ppargc1a* | AGT CCC ATA CAC AAC CGC AG | CCC TTG GGG TCA TTT GGT GA |
| *Tfam* | GGA ATG TGG AGC GTG CTA AAA | TGC TGG AAA AAC ACT TCG GAA TA |
| *mtDNA* | TCC TCT GAC AGG ATT GCA GC | CCG AGG GTG AAT GAC CAG AG |
| *Mfn* | AAC GCT CTC TCT TTC GCA CG | TTG GAA AAC AGT GGG CTG GA |
| *Drp1* | GCT GCC TCA GAT CGT CGT AG | GGT GAC CAC ACC AGT TCC TC |
| *Cpt1* | GGT CTT CTC GGG TCG AAA GC | TCC TCC CAC CAG TCA CTC AC |
| *Acox1* | CTT GGA TGG TAG TCC GGA GA | TGG CTT CGA GTG AGG AAG TT |
| *Hif1a* | GG ATG AGT TCT GAA CGT CGA AA | AAT ATG GCC CGT GCA GTG AA |
| *Glut1* | ACA CTC ACC ACG CTT TGG TC | ACA CAC CGA TGA TGA AGC GG |
| *Hk* | CCA AAA TAG ACG AGG CCG TA | TTC AGC AGC TTG ACC ACA TC |
| *Pkm1* | GCT GTT TGA AGA GCT TGT GC | TTA TAA GAG GCC TCC ACG CT |
| *Pkm2* | TCG CAT GCA GCA CCT GAT T | CCT CGA ATA GCT GCA AGT GGT A |
| *Ldha* | GCT CCC CAG AAC AAG ATT ACA G | TCG CCC TTG AGT TTG TCT TC |
| *Fn* | TGA CAA CTG CCG TAG ACC TGG | TAC TGG TTG TAG GTG TGG CCG |
| *Col1a1* | GCC AAG AAG ACA TCC CTG AA | GTT TCC ACG TCT CAC CAT TG |
| *Col1a3* | ACG TAA GCA CTG GTG GAC AG | CAG GAG GGC CAT AGC TGA AC |
| *Bax* | TGC AGA GGA TGA TTG CTGAC | GAT CAG CTC GGG CAC TTT AG |
| *Bcl-2* | AGG AGC AGG TGC CTA CAA GA | GCA TTT TCC CAC CAC TGT CT |
| *16s* | ACC ATG AAG ACC ATG ACA CAG G | TGT CTT CCT TTT TAA GGC TGT CAA |
| *18s* | CGC TTC CTT ACC TGG TTG AT | GGC CGT GCG TAC TTA GAC AT |

**Supplementary Table S2. Body weight, kidney weight, and biochemical parameters**

|  | ***db/m***  **(n=10)** | ***db/m* + TEPP-46**  **(n=10)** | ***db/db***  **(n=10)** | ***db/db* + TEPP-46**  **(n=10)** |
| --- | --- | --- | --- | --- |
| **Body weight, g** | 26.72 ± 2.18 | 25.69 ± 2.08 | 40.17 ± 2.74^*^ | 39.58 ± 3.48 |
| **Kidney weight, g** | 0.184± 0.016 | 0.186 ± 0.018 | 0.217 ± 0.020^*^ | 0.224 ± 0.021 |
| **Serum biochemistry** |  |  |  |  |
| Glucose, mg/dL | 141.2 ± 15.6 | 142.3 ± 17.9 | 550.0 ± 31.3^*^ | 534.8 ± 29.3 |
| Glycated hemoglobin mg/dL | 3.86 ± 0.18 | 3.70 ± 0.27 | 7.19 ± 0.70^*^ | 7.17 ± 0.42 |
| BUN, mg/dL | 22.0 ± 0.7 | 22.0 ± 1.1 | 34.8 ± 2.2^*^ | 26.6 ± 1.8^#^ |
| Creatinine, mg/dL | 0.16 ± 0.03 | 0.18 ± 0.07 | 0.45 ± 0.06^*^ | 0.34 ± 0.10^#^ |
| **24-h albuminuria, mg/day** | 17.8 ± 5.7 | 16.7 ± 6.1 | 203.1 ± 31.8^*^ | 111.3 ± 25.6^#^ |

Notes: Data are expressed as mean ± SD

*, *P*<0.05 vs. *db/m*; #, *P*<0.05 vs. *db/db*

**Supplementary Figures**

**Supplementary Figure S1. Blood glucose during the experiment period.** Five mice each in the diabetes and control groups were intraperitoneally injected with TEPP-46 (10 mg/kg) or dimethyl sulfoxide, respectively, at 6 weeks of age for 6 weeks.

**
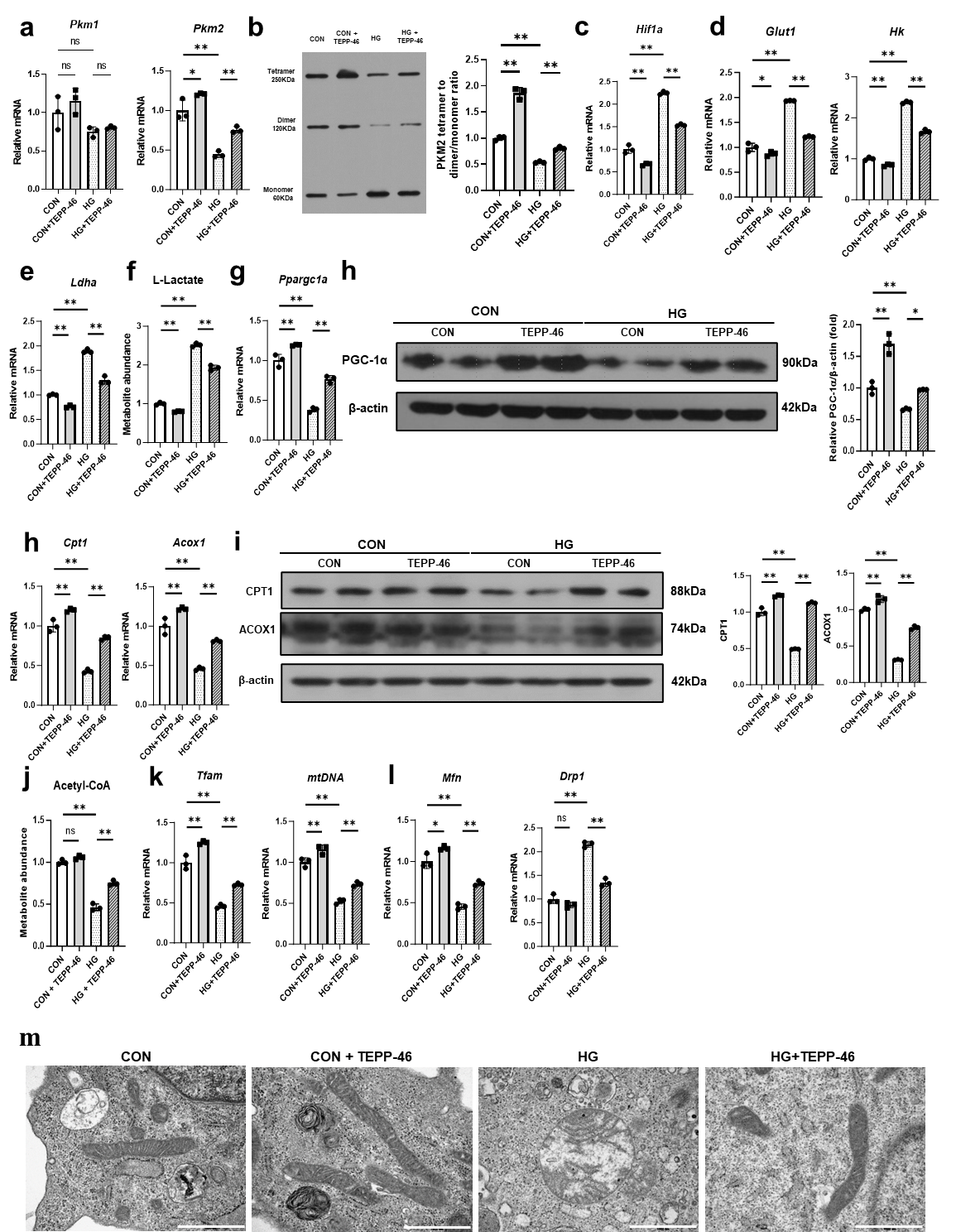
**

**
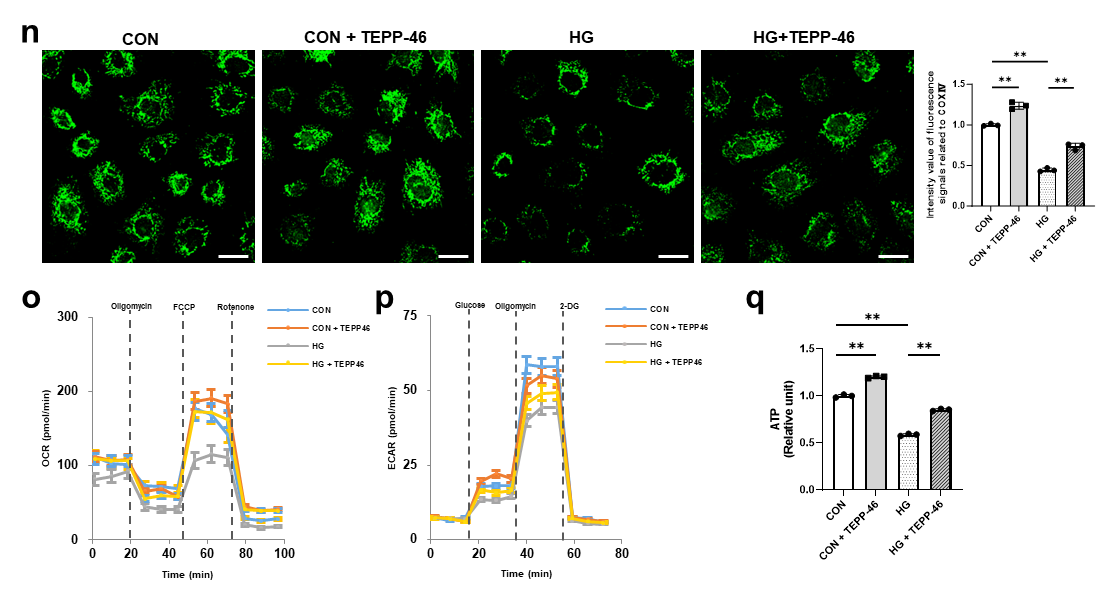
 Supplementary Figure S2.** **PKM2 activation by TEPP-46 treatment improves aberrant glycolytic flux and decreases PGC-1α expression and fatty acid oxidation in high glucose (HG)-treated renal tubular epithelial cells (RTECs).** Primary RTECs were stimulated with 40 mM glucose (HG) and 10 μM TEPP-46 for 48 h. (a) The mRNA expression levels of the *Pkm* isozyme were lower in HG-stimulated RTECs. (b) The ratios of the PKM2 tetramer to the dimer/monomer in a cross-linking study were lower in HG-stimulated RTECs, and PKM2 activity was restored by TEPP-46. (c-e) The mRNA expression levels involved in the glycolysis pathway were altered and (f) the concentrations of L-lactate were increased in HG-stimulated RTECs; this increase was reversed by co-treatment with TEPP-46. (g) The mRNA expression levels of *Ppargc1a* and the corresponding protein levels of PGC-1α were reduced in HG-treated RTECs, and these alterations were attenuated by TEPP-46 treatment. (h) The mRNA expression and (i) protein levels of CPT1 and ACOX1, markers of fatty acid oxidation, were reduced in RTECs treated with HG, and the reduction were reversed by TEPP-46 treatment. (j) The acetyl-CoA levels measured by a colorimetric assay were decreased in RTECs treated with HG, and the decreased levels were attenuated by TEPP-46 treatment. (k) The decreased mRNA expression levels of mitochondrial transcripts in RTECs with HG were restored by TEPP-46 treatment. (l) The mRNA transcript analysis of *Mfn* and *Drp1* showed a shift in mitochondrial dynamics toward mitochondrial fragmentation. These alterations were attenuated by TEPP-46 treatment. (m) Electron microscopic images of kidney mitochondria in RTECs with HG showed mitochondrial fragmentation and disrupted mitochondrial integrity. These alterations were attenuated by TEPP-46 treatment (scale bar = 2000 nm). (n) Representative sections of immunofluorescence staining for cytochrome c oxidate subunit 4 (COX IV) show a significant reduction in COX IV expression in HG-treated RTECs, which was attenuated by TEPP-46 treatment (scale bar = 20 μm). (o) HG-treated RTECs showed significant reductions in basal oxygen consumption rates, spare respiratory capacity, proton leak, and ATP production compared with controls. These alterations were reversed by TEPP-46 treatment. (p) Extracellular acidification rate (ECAR) was significantly reduced in HG-treated RTECs compared to controls, and was restored by TEPP-46 treatment. (q) ATP assays of RTECs measured by a colorimetric assay. HG-treated RTECs with HG exhibit low ATP concentration, and TEPP-46 treatment significantly restore ATP levels. For all groups, data are presented as mean ± SD (n = 3 per group). ns, *P* ≥0.05; *, *P* <0.05; **, *P* <0.01; CON, control.


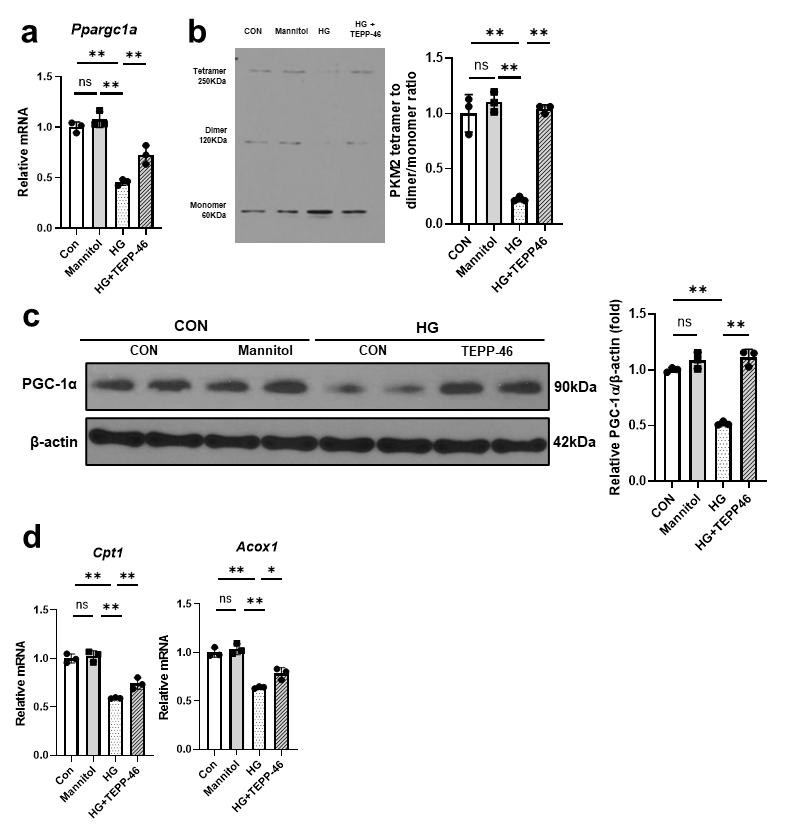


**Supplementary Figure S3. High glucose, not osmotic pressure, induces decreased PKM2 activation and PGC-1α expression in renal tubular epithelial cells.** Primary RTECs were stimulated with 40 mM glucose (HG) or 24 mM mannitol and HG+10 μM TEPP-46 for 48 h. (a) The mRNA expression levels of the *Pkm2* were similar with control in mannitol-stimulated RTECs. (b) The ratios of the PKM2 tetramer to the dimer/monomer in a cross-linking study were similar in mannitol-stimulated RTECs compared to control. (c) The PGC-1α protein levels are also comparable between control and mannitol-stimulated RTECs. (d) The mRNA expression levels of the *Cpt1* and *Acox1* were similar with control in mannitol-stimulated RTECs. For all groups, data are presented as mean ± SD (n = 3 per group). ns, *P* ≥0.05; *, *P* <0.05; **, *P* <0.01; CON, control.


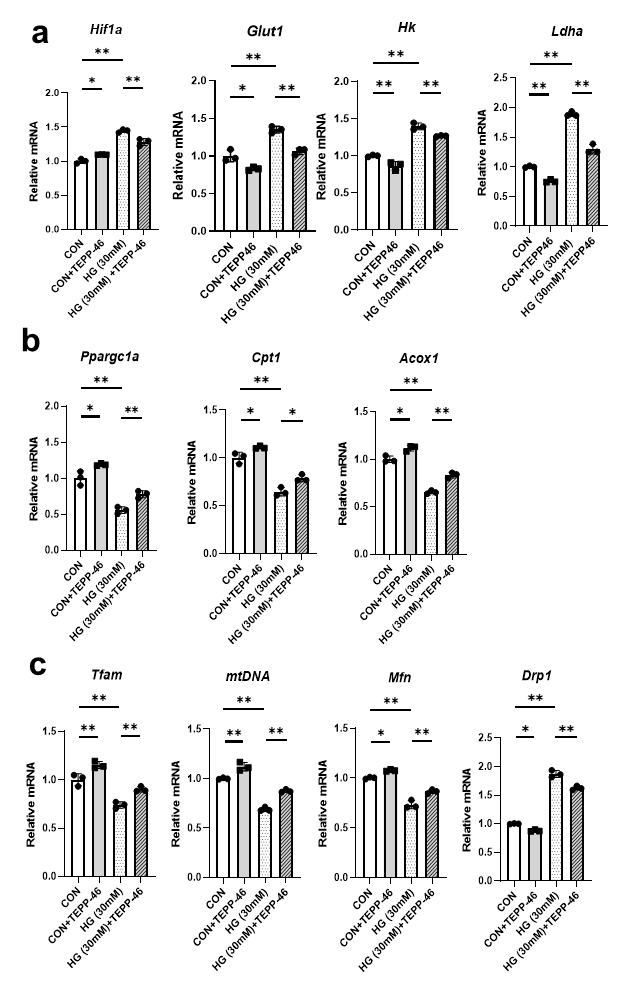


**Supplementary Figure S4 PKM2 activation by TEPP-46 treatment improves aberrant glycolytic flux, fatty acid oxidation, and mitochondrial dysfunction in 30 mM glucose-treated renal tubular epithelial cells (RTEC).** Primary RTECs were stimulated with 30 mM glucose (HG) and 10 μM TEPP-46 for 48 h. (a) The mRNA expression levels involved in the glycolysis pathway were altered in HG-stimulated RTECs; this change was reversed by co-treatment with TEPP-46. (b) The fatty acid oxidation related transcripts expression levels were reduced in HG-treated RTECs, and these alterations were attenuated by TEPP-46 treatment. (c) The decreased mRNA expression levels of mitochondrial mass and dynamics were altered in HG-stimulated RTECs. These alterations were attenuated by TEPP-46 treatment. For all groups, data are presented as mean ± SD (n = 3 per group). ns, P ≥0.05; *, P <0.05; **, P <0.01; CON, control.


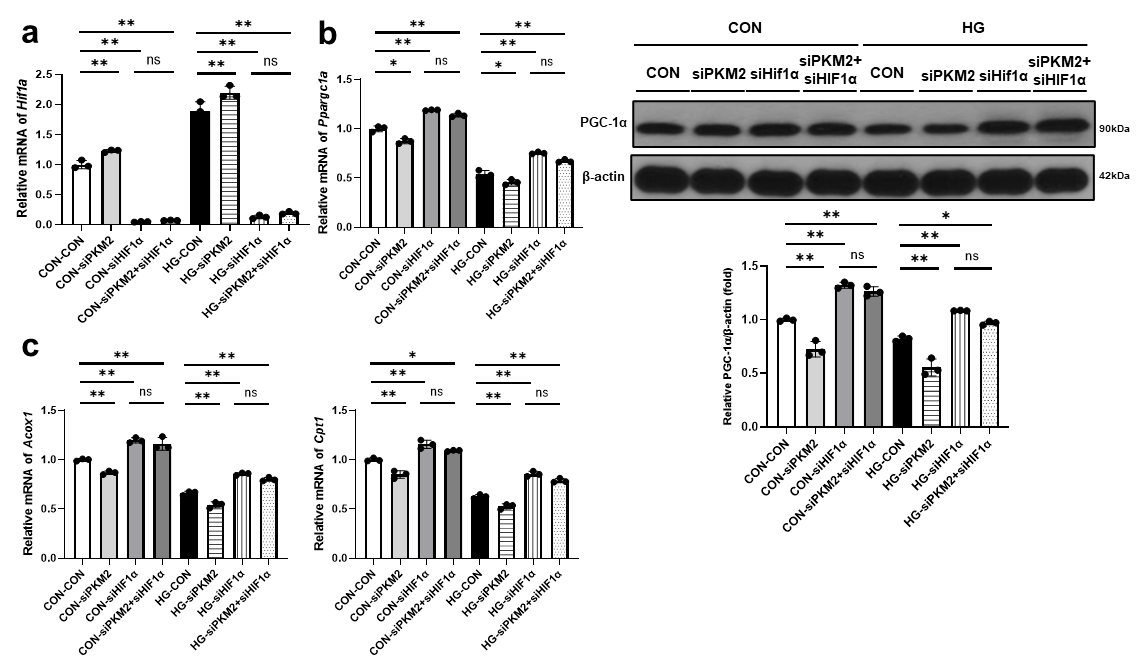


**Supplementary Figure S5. PKM2 regulates HIF-1α expression and PGC-1α activity in renal tubular epithelial cells (RTECs).** Primary RTECs were transfected with an *Pkm2* small interfering RNA (siRNA) plasmid (siPKM2), *Hif1a si*RNA plasmid (siHIF1α), siPKM2+ siHIF1α, or vector plasmid and 40 mM glucose for 48 h. (a) The expression level of *Hif1a* was increased in *Pkm2*-supressed RTECs in control group. These changes were amplified in HG-treated RTECs. The *Hif1a* mRNA expression levels were comparable between the siHIF1α-only-treated group and both siPKM2 and siHIF1α-treated, regardless of glucose condition. (b) In the control glucose group, mRNA expression and protein levels of PGC-1α decreased in siPKM2 treated-RTECs and increased in *Hif1a* silencing in control group. Under HG conditions, the mRNA expression level of PGC-1α decreased further in siPKM2-treated RTECs compared to HG-only treated RTECs. Silencing *Hif1a* reversed mRNA expression and protein levels of PGC-1α. (c) In the control glucose group, mRNA expression of *Cpt1* and *Acox1* decreased in siPKM2 treated-RTECs but increased in *Hif1a* silencing in control group. Under HG conditions, the mRNA expression of *Cpt1* and *Acox1* was further reduced in siPKM2-treated RTECs compared to HG-only RTECs. These changes were reversed by *Hif1a* suppression. The mRNA expression of *Cpt1* and *Acox1* were comparable between siHIF1α only treated-RTECs and both siHIF1α and siPKM2 treated RTECs. For all groups, data are presented as mean ± SD (n = 3 per group). ns, *P* ≥0.05; *, *P* <0.05; **, *P* <0.01; CON, control.

**Supplementary Figure S6. PKM2 regulates PGC-1α activity via both HIF-1α–dependent and –independent mechanisms.** Primary RTECs were treated for 48 h under the following conditions: control (normal glucose), high glucose (HG, 40 mM), HG with *Hif1a* siRNA (siHIF1α), HG with TEPP-46 (10 μM), and HG with both siHIF1α and TEPP-46. TEPP-46–mediated PKM2 activation restored *Ppargc1a* expression under HG, and this effect persisted in part following HIF-1α silencing, indicating a HIF-1α–independent component in PKM2-mediated PGC-1α regulation. For all groups, data are presented as mean ± SD (n = 3 per group). ns, *P* ≥0.05; *, *P* <0.05; **, *P* <0.01; CON, control.


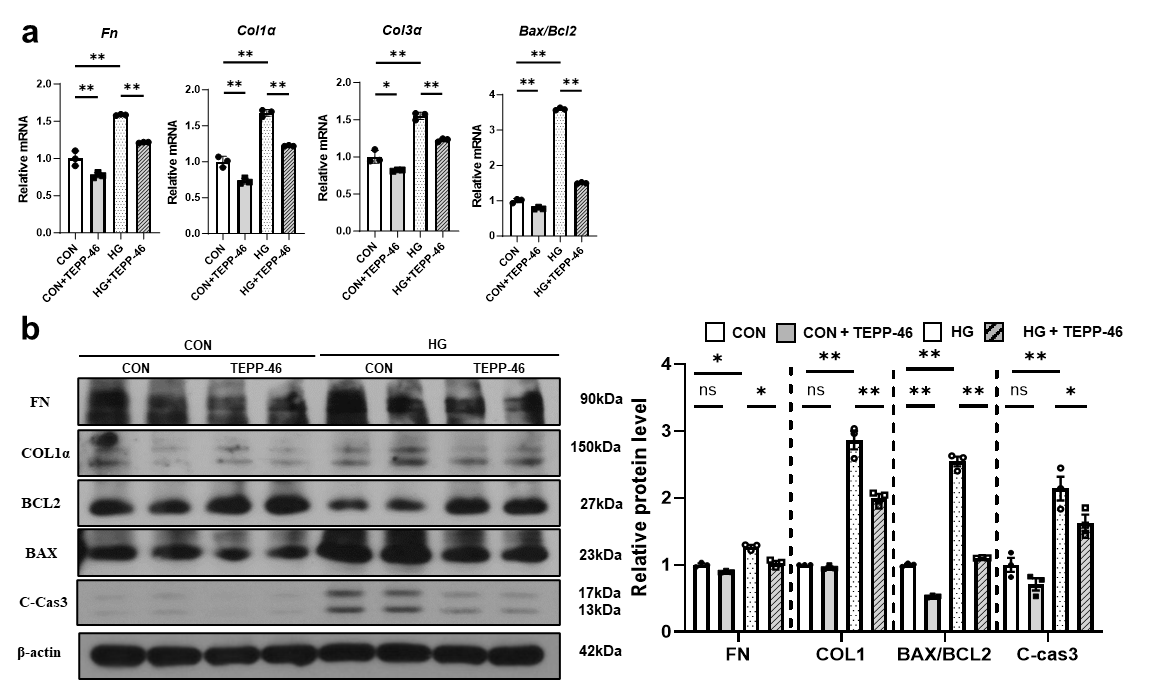


**Supplementary Figure S7. PKM2 activation by TEPP-46 treatment restores kidney fibrosis and apoptosis in high glucose (HG)-treated renal tubular epithelial cells (RTECs).** Primary RTECs were stimulated with 40 mM glucose (HG) and 10 μM TEPP-46 for 48 h. (a) The mRNA and (b) protein expression levels of pro-fibrotic markers and apoptotic cell death markers were increased in HG-treated RTECs compared with the control group, and this alteration was attenuated upon TEPP-46 treatment. For all groups, data are presented as mean ± SD (n = 3 per group). ns, *P* ≥0.05; *, *P* <0.05; **, *P* <0.01; CON, control.

**
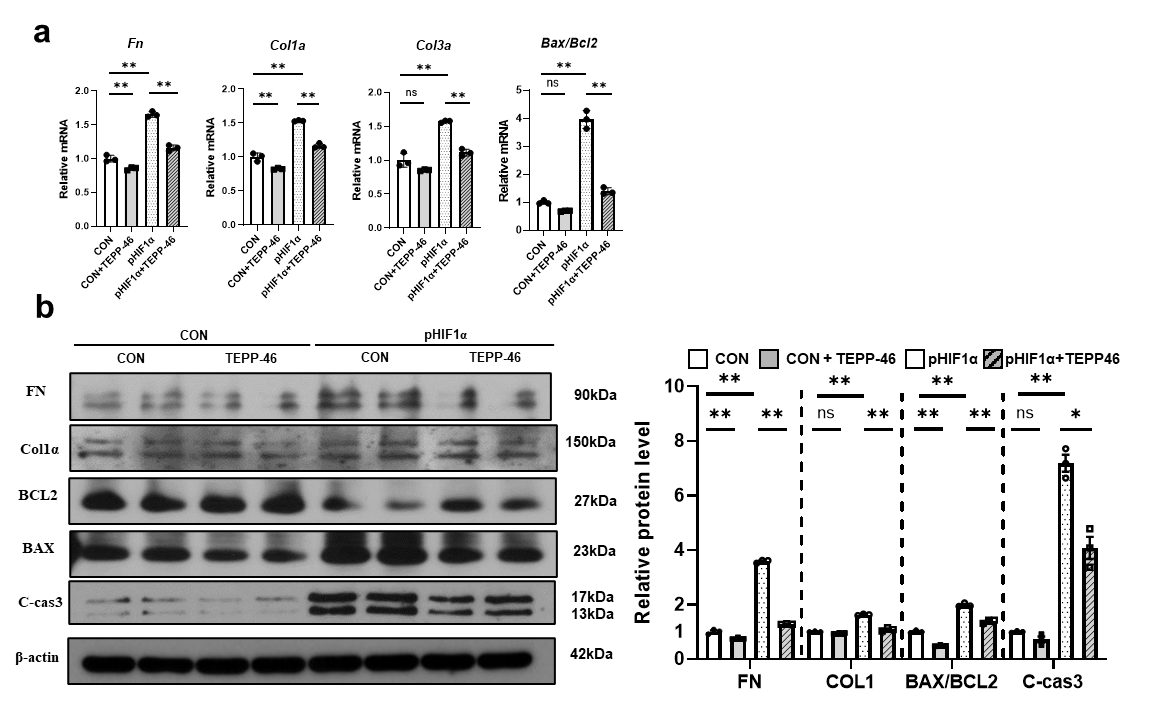
**

**Supplementary Figure S8. PKM2 activation by TEPP-46 treatment restores kidney fibrosis and apoptosis in HIF-1α-overexpressing renal tubular epithelial cells (RTECs).** Primary RTECs were transfected with the *Hif1a* plasmid (pHIF1α) or vector plasmid and 10 mM TEPP-46 for 48 h. (a) The mRNA and (b) protein expression levels of pro-fibrotic markers and apoptotic cell death markers were increased in pHIF1α-transfected RTECs compared with controls, and this alteration was attenuated upon TEPP-46 treatment. For all groups, data are presented as mean ± SD (n = 3 per group). ns, *P* ≥0.05; *, *P* <0.05; **, *P* <0.01; CON, control.


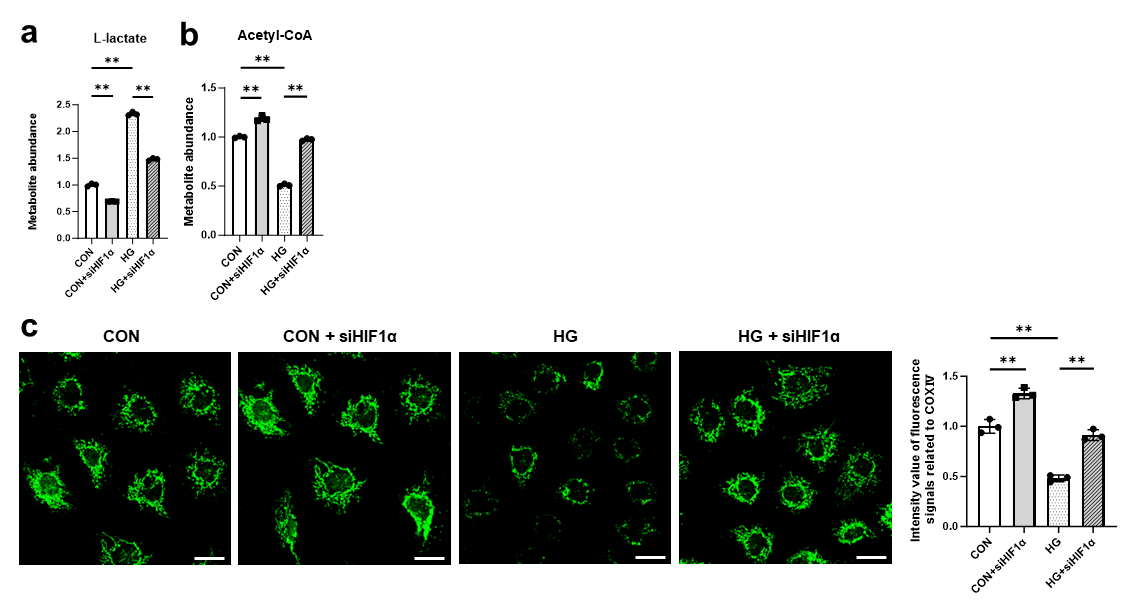


**Supplementary Figure S9. Silencing *Hif1a* reverses altered energy metabolism and mitochondrial dysfunction in high glucose (HG)-treated renal tubular epithelial cells (RTECs).** Primary RTECs were transfected with an *Hif1a* small interfering RNA (siHIF1α) plasmid or vector plasmid and 40 mM glucose for 48 h. (a) The concentration of L-lactate was increased in HG-treated RTECs. These changes were reversed by transfection of the siHIF1α plasmid. (b) Acetyl-CoA levels measured by the colorimetric assay was decreased in HG-treated RTECs, and this alteration was restored by transfection with the siHIF1α plasmid. (c) Representative sections of immunofluorescence staining for cytochrome c oxidate subunit 4 (COX IV) showed a significant decrease in COX IV intensity in HG-treated RTECs, and this alteration was reversed by transfection with the siHIF1α plasmid (scale bar = 20 μm). For all groups, data are presented as mean ± SD (n = 3 per group). ns, *P* ≥0.05; *, *P* <0.05; **, *P* <0.01; CON, control.
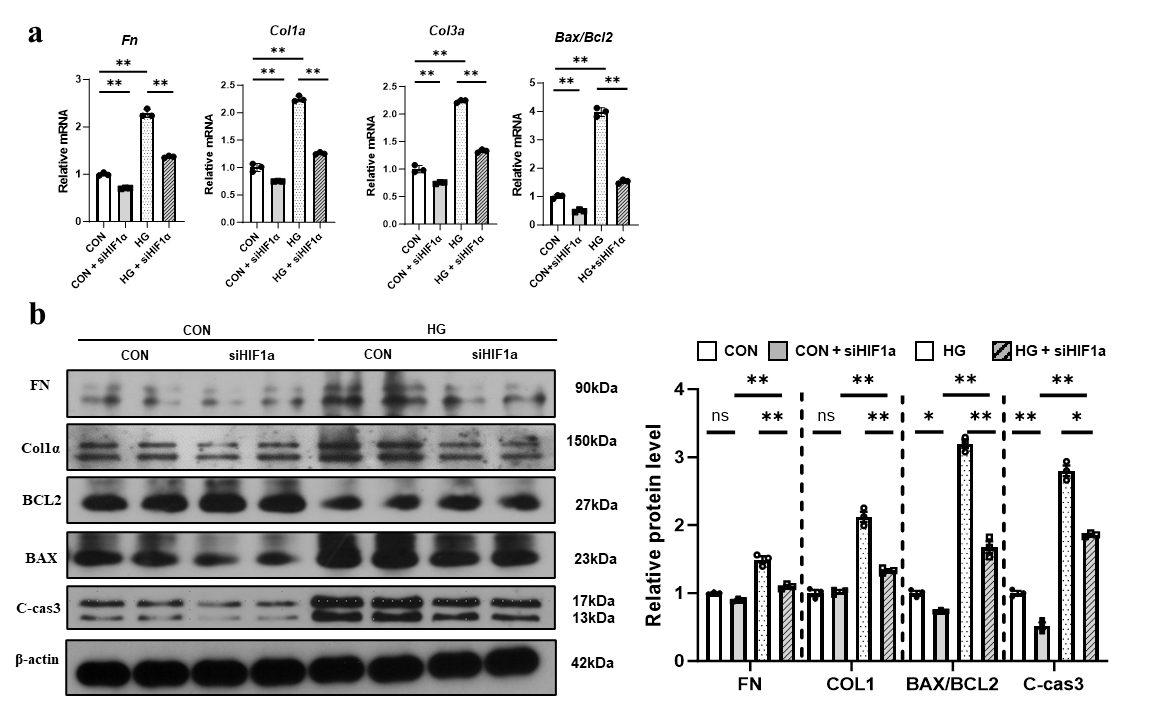


**Supplementary Figure S10.** **Silencing *Hif1a* decreases the expression of fibrotic markers and apoptosis in high glucose (HG)-treated renal tubular epithelial cells (RTECs)**. Primary RTECs were transfected with the *Hif1a* small interfering RNA (siHIF1α) plasmid or vector plasmid and 40 mM glucose for 48 h. (a) The mRNA and (b) protein expression levels of pro-fibrotic markers and apoptotic cell death markers were increased in HG-treated RTECs compared with controls, and this alteration was attenuated with *Hif1a* silencing. For all groups, data are presented as mean ± SD (n = 3 per group). ns, *P* ≥0.05; *, *P* <0.05; **, *P* <0.01; CON, control.
